# How Complexity and Uncertainty Grew with Algorithmic Trading

**DOI:** 10.3390/e22050499

**Published:** 2020-04-26

**Authors:** Martin Hilbert, David Darmon

**Affiliations:** 1 Communication, Computational Social Science, University of California, Davis, CA 95616, USA; 2 Department of Mathematics, Monmouth University, West Long Branch, NJ 07764, USA; ddarmon@monmouth.edu

**Keywords:** algorithmic trading, complexity, predictability, machine learning, dynamical systems theory

## Abstract

The machine-learning paradigm promises traders to reduce uncertainty through better predictions done by ever more complex algorithms. We ask about detectable results of both uncertainty and complexity at the aggregated market level. We analyzed almost one billion trades of eight currency pairs (2007–2017) and show that increased algorithmic trading is associated with more complex subsequences and more predictable structures in bid-ask spreads. However, algorithmic involvement is also associated with more future uncertainty, which seems contradictory, at first sight. On the micro-level, traders employ algorithms to reduce their local uncertainty by creating more complex algorithmic patterns. This entails more predictable structure and more complexity. On the macro-level, the increased overall complexity implies more combinatorial possibilities, and therefore, more uncertainty about the future. The chain rule of entropy reveals that uncertainty has been reduced when trading on the level of the fourth digit behind the dollar, while new uncertainty started to arise at the fifth digit behind the dollar (aka ‘pip-trading’). In short, our information theoretic analysis helps us to clarify that the seeming contradiction between decreased uncertainty on the micro-level and increased uncertainty on the macro-level is the result of the inherent relationship between complexity and uncertainty.

## 1. Introduction

The information revolution has not only revolutionized business, the economy, politics, and socio-cultural conduct, but also the *modus operandi* of financial markets. Looking at the hustling and bustling that was still ongoing on trading floors only a decade ago, and comparing them to the smooth humming of today’s computational trading floors, suggests that algorithmic trading has had a major effect on the way financial assets change hands. In this study, we look for observable signatures that evidence changes in the nature of trading dynamics over the last decade. We find that the overall emergent trading dynamic became more predictable, more complex, and more uncertain at the same time. We choose the foreign exchange market for our analysis, since it has experienced clear and delineable growth of algorithmic trading.

In general, traders employ algorithmic automation to make their local dynamics more reliable and predictable. A large literature also shows that it makes trading faster, but in this study, we are not concerned with this aspect. Precision of prediction is the name of the game of the currently dominating machine learning paradigm [1]. Most digitally automated information processes, including bots, trading algorithms, and all kinds of artificial intelligence (AI), follow a set of deterministic local rules that either respond to programmed instructions or learned patterns. For example, a foreign exchange algorithm can buy or sell as response to a set of input, which can either be predefined (so-called expert systems), or self-learned (so-called machine learning). The algorithm will reliable and predictably buy or sell according to the current version of the step-by-step recipe. Algorithms are defined as “an ordered set of unambiguous, executable steps that defines a terminating process” [2]. Therefore, per definition, algorithms predictably execute a (given or self-learned) recipe in order to reach an inevitable conclusion that defines their behavior deterministically.

In our analysis, we do find evidence that the employed algorithmic machinery in foreign exchange markets is associated with an additional level of predictable structure to the evolving dynamics of bid-ask spreads. Intricate new subsequences provide the dynamic with an increase in predictable complexity. We show that algorithmic trading is one of the main explanatory variable for this tendency. We also show that algorithmic trading correlates with increased predictability, because it reduces the future uncertainty about the next bid-ask spread. However, this only holds if we look at the dynamics from the level of detail on which trade happened a decade ago. Putting on the coarse-grained lenses traders used to look at reality a decade ago, algorithms squeezed out all uncertainty from the market. There is no profit anymore trading on the fourth digit behind the dollar. Algorithms replaced surprise with predictable structure (more complex, but more predictable).

At the same time, the last decade has also seen an augmented level of fine-graining in trading dynamics. Algorithms were used to exploit a more detailed level of reality, where humans do not reach. At this new tick-trading level, we find an unprecedented amount of both predictable complexity and unpredictability at the same time. From the perspective of the fifths digit behind the dollar (where profits are made nowadays), uncertainty is larger than ever before. The algorithmification of foreign exchange trading is linked to the reduction of uncertainty as it was the norm a decade ago, but also to a new and more detailed outlook on reality, which is much more uncertain.

Our findings suggest that traders introduce automated algorithms to make their daily routines more predictable and that they would have been successful in taming the markets if the world would have stayed on the coarse-grained black and white level it used to be at. However, by doing this, they opened up a new level of unprecedented shades of grey and ended up making the entire market system less predictable than ever before.

The paper proceeds as follows. Based on existing literature, we formulate two complementary hypotheses regarding changing trade dynamics in foreign exchange markets. We then propose to quantify market dynamics with the measurement apparatus from dynamical systems theory and present three longstanding and complementary measures. We obtain almost a billion tick-level trades from eight currency pairs for the eleven years between 2007 and 2017 and calculate the proposed measures for 528 bimonthly periods. We then use straightforward multiple linear regression analysis to test our hypotheses regarding the increasing role of algorithms and our signatures of changing trade dynamics. Finally, we interpret our findings with the help of existing theorems and literature from information-theoretic approaches to dynamical systems. Our results showcase a methodology to assess dynamical changes provoked by algorithms and contribute to ongoing discussions about the effect of ever more sophisticated algorithmic trading on market dynamics.

## 2. The Rise of Algorithmic Trading

Trading markets have been called “the world’s largest and most powerful techno-social system” [3]. The main driver of technological change over the last decade has consisted in the introduction of algorithmic trading (AT). AT can be broadly defined as a set of automated trading strategies that follow certain variables in their decision-making process, such as time, price, and volume, and other historical and simulated patterns.

Farmer and Skouras [4] distinguish among three broad groups of trading algorithms. The first are execution algorithms (often referred to as ‘algos’ in the literature). They consist of instructions that allow humans to set the parameters for trade execution, such as a determined timeframe, volume patterns, risk-adjusted real-time market conditions, relative prices between selected stocks, etc. Their foremost goal is not necessarily to make an extra trading profit, but to minimize cost and risk, and to assure reliability in the execution of an order according to a set strategy. As straightforward as ‘execution algos’ are, they automate many different aspects of trading. A decade ago, an investor trying to buy a sizable amount of stocks had to hire a floor broker to quietly work the order, using human judgment to buy bits and pieces of the total trade to avoid driving the share price up. Execution algorithms can buy or sell on the timescale of days, or even months. Many of them use machine learning to understand market patterns.

The second group refers to the notorious high-frequency trading (HFT). The focus is set on a high volume order-to-trade ratio [5]. A popular strategy consists in taking advantage of small differences before others, and creating scale by volume and frequency. In the lingo of some of the related literature, HFT often uses a “sub-penny jumping strategy to front-run manual traders” [6].

The third big group refers to algorithmic market making. The classic historical case of market making is a mediator at a travel hub who buys and sells currencies to travelers who arrive and leave a certain country. The difference obtained from temporal and spatial asymmetries can be thought of as a fee for the liquidity the market maker provides. In other words, market makers take advantage of the fact that supply and demand are not equal everywhere and for everybody all the time. Algorithmic search lends itself naturally to identify such asymmetries, as it “increases the amount of price discovery that occurs without trading, implying that quotes become more informative” [7]. Some argue that HFT are the new market maker, as both often show up together [8].

Combining this diversity of trading algorithms with their various degrees of automation makes it tricky to measure their growing participation in trading activity consistently. The literature reports initial influence of algorithmic trading as far back as the mid-1990s [9]. In 2003, the New York Stock Exchange introduced ‘autoquote’, an automated system that disseminates a new quote automatically, catering to algorithmic trading [7]. Several markets provided ‘automated interfaces’ or ‘automatic electronic trading systems’ since the mid-2005. However, both also include orders received from humans that are executed at the very last step by a machine. Therefore, there are different definitions related to the level of automation. Even with existing electronic trading infrastructures in use, there is evidence that the level of sophistication of algorithmic trading has increased notably thereafter. Different studies measure different variables to show this. Private sector sources estimate that IT spending on the sell-side of FX markets has stayed constant between 2008 and 2013, while the IT spending on the buy-side (where the main benefit of trading algorithms is found) is estimated to have multiplied by a factor of 2.5 [10]. For FX markets in 2011, Schmidt (2012) found that manual traders account for approximately 75% of all customers in EUR/USD, but they only placed 3.7% of daily orders, while ‘ultra-HFT’ represented 61.6% of daily orders. Measuring the number of US$ spent, or the number of traders, or the number of trades leads to very different levels of ‘algorithmic participation’.

**Figure 1:**
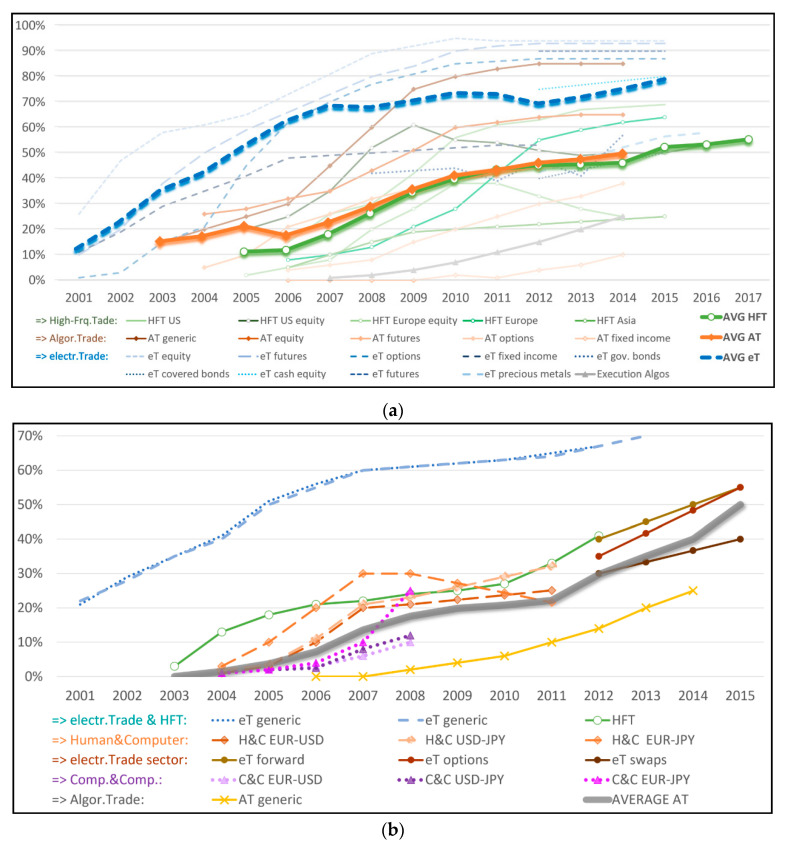
Participation of algorithmic trading of different kind in different markets. (**a**) General Estimates; (**b**) Foreign Exchange (FX). Sources: (**a**) [9,10,11,12,13,14,15,16,17,18,19] (**b**) [9,11,12,13,15,18,19,20].

With all of these differences in mind, Figure 1a provides a rough overview of several estimates of the pervasiveness of algorithmic trading in different markets. The blue dashed line represents the average growth tendencies of the estimated penetration of what is referred to as ‘electronic trade’, the version with a relatively vaguely defined technological component. The green-circle line represents the average of estimated participation of ‘high-frequency trading’, and the orange-diamond line the average of what literature refers to as ‘algorithmic trading’. Note that even these categories are not consistently defined. The figure gives an idea of the general growth tendencies and their approximate timing. It shows that notable and monotone growth of algorithmic participation has taken place over more than a decade.

Having studied the rise of algorithmic trading in different markets, we decided to focus our analysis on the foreign exchange (FX) market. Not only is the FX market the largest financial market in the world that affects growth, inflation, interest rates, unemployment, and international relations, but it also provides for a nice case study of the role of algorithms since they arrived to FX relatively late. As suggested by the data gathered in Figure 1b, electronic interfaces were quite ubiquitous in FX by 2007, but more sophisticated forms of AT have entered the market later, after the growth of less sophisticated forms had started to stagnate. Probably the most reliable and best documented data comes from the analysis of Chaboud et al. [9] and Schmidt [11] with data from the FX platform Electronic Broking Services (EBS). Chaboud et al. [9] identify those trades where computers are both taker and maker of the trade and measure that in 2007 the respective fractions of trade volume was at 5% for EUR/USD and USD/JPY and 11% for EUR/JPY, both doubling the following year, respectively.

We, therefore, decided to make 2007 the starting year of our analysis. Most sources agree that during the subsequent decade, algorithmic trading started to play a prominent role in FX markets, which suggests that we should be able to identify some empirical signatures of their role in market dynamics. It is important to note that the literature argues that human traders will continue to play an essential role in FX trading because of their safeguard function in case of unexpected shocks [21]. Schmidt [11] found that large orders usually involve manual traders and algorithms alone, practically never used order sizes of more than EUR 3–4 million. However, Figure 1b suggests important growth of AT in FX markets over the decade since 2007.

## 3. Hypothesized Effects

Going back to the first pioneering studies on algorithmic trading, it has become clear that digital “agents consistently obtain significantly larger gains from trade than their human counterparts” [22]. All three of the aforementioned groups of AT contribute to this, including execution algorithms [23], quickly reacting algorithms [7], and algorithmic market making [9]. We, therefore, frame market dynamics in terms of the evolving bid-ask spread, a primary indicator for the obtainable margin.

The bid-ask spread is the difference between the highest price that a buyer is willing to pay for an asset and the lowest price that a seller is willing to accept to sell it. In what is known in the literature as a continuous double auction (CDA), buyers announce bid-prices at any time, and sellers are free to ask for different offer-prices. At any time, any seller is free to ‘hit’ any bid. If someone wants to buy a Euro for 1.10 dollars but ends up accepting a selling offer of 1.13 dollars, the bid-ask spread is 0.03. The electronic order book will typically show quote data in four columns or lists, for bids and for asks, and for the quantity available at that price. Every hour of every working day, trillions of dollars-worth of orders flow through CDA markets. As such, the bid-ask spread is the main guidepost to show how closely demand and supply are in equilibrium.

In the Supporting Information, i.e., SI.7, we analyze the question if the bid-ask spread has decreased over the past decade. In agreement with the literature on this question [6,8,11,24,25], we also find evidence for this in our dataset.

The spread is traded in ‘ticks’. A tick denotes a market’s smallest possible price movement to the right of the decimal, and in the currency market, it is often also referred to as pip (point in percentage). It varies depending on how a given currency pair is traded [6]. In March 2011, the electronic broking service (EBS) decided to reduce the tick size by a factor ten (known in the literature as a transition from pip to decimal pip). When the decimal level was fine-grained to the next more detailed decimal, the literature talks about decimal pip pricing. The reinforcing positive feedback loop between increasing decimalization and algorithmic trading is taken as a well-known fact in the literature [26].

It is important to note that the focus here is set on a refinement in the detail of a currency, not in the speed of its execution. After 2006, some stock exchanges, among them the New York Stock Exchange, increased automation (hybrid market), which reduced the execution time for market orders from 10 s to less than one second and by itself, did not provide notable changes to the bid-ask spread [27]. It is reported that EBS mainly made this shift to make the market more attractive to algorithmic HFTs [6].

### 3.1. Complexity (H1)

From a perspective of economic theory, trading algorithms provide a clear example of bounded rationality [28]. Bounded rationality means that individual algorithms are limited by the information they have, by their processing capacities and biases, and by the finite amount of time they have to make a decision (pushed by others in the market). Algorithms are highly specialized decision-making rules. This is actually necessary by a fundamental mathematical theorem, known as the ‘no-free-lunch theorem’ [29], which establishes “that for any algorithm, any elevated performance over one class of problems is offset by performance over another class” [30]. This in turn implies that to perform all functions demanded of trading algorithms, the universe of their collective is necessarily highly diverse. “Consequently the universe of computer algorithms is best understood as a complex ecology of highly specialized, highly diverse, and strongly interacting agents” [4]. There is increasing evidence that “even relatively ‘dumb’ bots may give rise to complex interactions”, produced by recursive interactions, fights, disagreements and conflicts [31]. Additionally to the arising diversity, each entity has also become more complex. “Computers have driven the creation and trading of increasingly complex financial products” [4], which, together with “the extreme complexity of the interaction of effects… and the multi-platform nature of the economics of market microstructure” [4] is reminiscent of a highly complex ecosystem. Together, these interactions “can generate a new behavioural regime” [3]. This would suggest that algorithmic trading correlates with more complex market dynamics.

At the same time, more quantitative analysis has found the opposite: less complexity. Park, Won Lee, Yang, Jo, & Moon [32] analyzed the S&P500 between 1983 and 2006 and concluded that increased information flow led to shorter and simpler patterns. Chaboud et al. [9] find a decrease in the degree of autocorrelation in algorithm-to-algorithm trades, as they seem to post quotes that reflect new information more quickly. Reduced autocorrelation would mean a less complex sequence since there are fewer temporal signatures that give some kind of recognizable rhythm to the series of events. For mixed trades of algorithms with humans, their evidence is not as clear [9]. While we will have to explain these contradictory empirical results, we follow the rather qualitative reasoning presented here and expect ever more complex algorithms giving rise to more complex market dynamics, which we make our first hypothesis (H1):

**Hypothesis** **1** **(H1).** *The involvement of algorithms is associated with more complex trading dynamics.*

### 3.2. Uncertainty (H2)

Technological innovation in trading is driven by a billion-dollar arms race to make algorithms both faster respondents and better predictors. Ever since the pioneering economist Léon Walras conceptualized the process of matching supply and demand through consecutive trial and error in the late 1800s (*tâtonnement* in his native French), market strategists have employed ever more sophisticated tools to accelerate and eradicate this mismatch. Machine-learning powered trading algorithms are the most recent culmination of these efforts, as they embody the automation of this search process of trial and error to match supply and demand. With better predictions, they end up needing fewer trials and fewer errors and close the bid-ask spread.

Therefore, asking about the effect of trading algorithms on predictability seems almost tautological, since today’s artificial intelligence is all about predictions. “The current wave of advances in artificial intelligence doesn’t bring us intelligence but instead a critical component of intelligence: prediction” [1]. Today’s AI is mostly equivalent with machine learning [33], and machine learning aims at picking up patterns from data (and data is always from the past, [34,35]) and using it to make predictions about the future. Trading algorithms that are better at predicting market asymmetries and margins are more profitable.

Chaboud et al. [9] found that algorithmic trading activity in the FX market reduces the number of triangular arbitrage opportunities, as algorithmic traders quickly respond to the posted quotes by non-algorithmic traders and profit from any potential arbitrage, which might be an indicator for better predictions. Cvitanic & Kirilenko [36] model an electronic limit order market and find that after the entry of an algorithmic high frequency trader, the distribution of transaction prices has more mass around the center and thinner far tails. They show that the faster humans submit and vary their orders, the more profit the machine makes, as they could better predict the dynamics. This leads to our second hypothesis (H2):

**Hypothesis** **2** **(H2).** *Algorithmic trading is associated with less uncertain and more predictable bid-ask spreads.*

## 4. Data

We use a series of independent predictor variables that characterize general economic tendencies that potentially affect FX trading, with the level of algorithmic trading as our focus of interest. As for the dependent response variable, we use three summary statistics of the degree of structure and randomness in the bid-ask spread, which we derive from dynamical system theory.

### 4.1. Dependent Variables

Our main data comes from Swiss FX Marketplace (SWFX), the electronic communications network (ECN) proprietary technological solution of Dukascopy Bank, launched in 2006. In contrast to EBS (another popular source in the literature), it is not a platform for market-making banks but has clients from the retail sector. Among the ten FX marketplaces in the world, it has a unique position by combining liquidity of the biggest marketplaces and banks. It is specialized in institutional liquidity and instant execution. Its JForex platform is available for traders interested in manual and automated trading. We obtain the data through Dukascopy Bank, which gives access to the Swiss Forex Marketplace [37]. Tick data of executed bid and asks are recorded at the millisecond, so for example, at 23:59:59.963, which determined the sequences order of our dynamics.

One advantage of this source, as compared to others, is that an ad-hoc change in the system does not confound trading granularity. In March 2011, EBS decided to reduce the tick size by a factor ten (known as a transition from pip to decimal pip), aiming at making the market more attractive to algorithmic HFTs [6], but soon after that gave in to protests from manual traders and revised it to half-pip pricing for most currency pairs in September 2012 (half a pip is special decimal pip pricing in which traders can use only 0 or 5 as the final digit). These events imply natural breaking points in the related time series when analyzing the dynamics, and are therefore less adequate for our purposes. Our source SWFX did not offer half-pip prices at any point in time but transferred straight to decimals early on [38]. Our data shows that decimal pricing was already in place in 2007, which gives us comparable longitudinal data back to that year.

In total, for the eleven years between January 1, 2007 and December 31, 2017, our source provided 2,251,845,424 trades on the tick level, for eight currently pairs, namely AUD/JPY, EUR/AUD, EUR/GBP, EUR/JPY, EUR/USD, GBP/JPY, GBP/USD, and USD/JPY. To obtain comparable dynamical systems measures, the longitudinal sample sequences need to be of the same sample length (shorter/longer sequences affect the sample likelihood of containing less/more diversity in the patterns). After analyzing the available data, we decided to join bi-monthly sequences into one single period (average of 4,264,859 trades per two months). We analyzed the first 1,750,000 consecutive trades of each bi-monthly period. This gave us 528 comparable datasets (8 currencies * 6 bi-monthly periods per year * 11 years), with a total of 924,000,000 data points.

This choice was made after considering the trade-off between sample sizes within and among time sequences. The length of the available time series affects the number of consecutive terms (unit of dynamic analysis), as well as the choice of the number of bins, since it has to be assured that each distinction among different realizations of a categorical variable provides representative statistics. A longitudinal sequence of T consecutive symbols, which consists of A different realizations of each symbol (the size of the alphabet, or the cardinality of the realizations of the random variable), in which each group is analyzed up to a window length L = 2, on average provides n samples per word (captured by the window length: $\frac{T}{n}=A^{L}$. This is equation gives a lower bound for expected sample size [39].

It is important to emphasize that our analysis works with sequence data, not considering time stamps. When pondering hypothesized effects of algorithmic trading, the first thing that might have come to mind might have been speed, which is an aspect that has gained much visibility with high-frequency trading [40,41]. In this study, we do not focus on the notorious refinements of reaction times, but rather on any changes in the refinement in currency details. In other words, we focus not on the granularity in time, but rather on the granularity in space, and its more or less uncertain distributions over the different trading options. Uncertainty, predictability, and complexity refer to the question of what is the next trade, not when it will happen. This decision implies that we work with sequence data, without coding for the length of the period between consecutive trades.

We then bin the bid-ask spread data, to be able to work with the more straightforward categorical variables used in discrete information theory (as compared to the more involved measures from continuous information theory) [42]. First, we bin the largest 1% of spreads per currency into a single bin, which avoids giving too much weight to extreme outliers (For all currencies, the largest 1% of spread is beyond 3 times the interquartile range (IQR), which is conventionally considered as “extreme outliers”. IQR multipliers for 1% cut-off: AUD/JPY: 3.2; EUR/AUD: 5.4; EUR/GBP: 5.2; EUR/JPY: 3.0; EUR/USD: 3.2; GBP/JPY: 4.1; GBP/USD: 3.3; USD/JPY: 3.5). Then we bin the data into both 200 bins (which we denote with the subscript [__200_]) and then merge ten consecutive bins on a higher level, to get another more coarse-grained binning with 20 bins (which we denote with the subscript [__20_]). One can think of the more fine-grained setup as trading on one more digit behind the dollar (e.g., distinguishing between a hundredth or a thousand of a cent).

We calculate three complementary information theoretic measures that characterize a dynamical process, all measured in bits (see also [43]). These measures are commonly used in the natural sciences and in dynamical systems theory, and go back to the time shortly after Claude Shannon [44] conceptualized information theory. Led by the prominent probability theorist Andrey Kolmogorov [45,46,47], information theory has long been used to quantify the inherent uncertainty (or predictability) of dynamical systems. More recent expansions into the field of theoretical computer science have added complementary measures [48,49,50,51]. This widely used mathematical framework comes with many advantages in the study of complex dynamics, most importantly that it naturally picks up nonlinear multiway interactions just as easily as simple pairwise linear correlations. In general, the corresponding measures provide summaries of the dynamical characteristics of the underlying temporal sequence.

**Figure 2:**
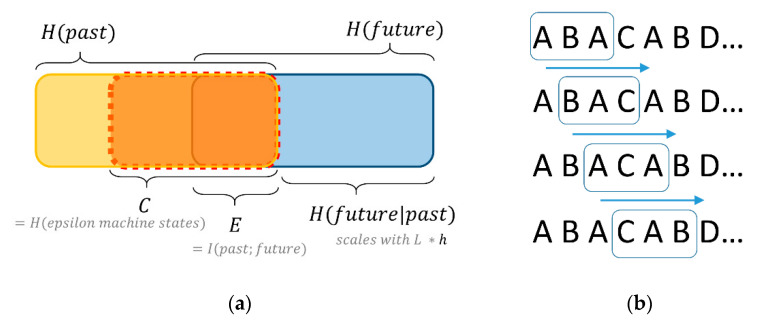
(**a**) Info-diagram of entropies of past (*H*(*past*)) and future (*H*(*future*)), relating predictive complexity (*C*), predictable information (*E*), and remaining uncertainty (h * L = *H(future|past)*); (**b**) schematic illustration of how to obtain statistics from a sequence consisting of a categorical variable with four different bins (A, B, C, D) by employing a sliding window of length *L* = 3.

The complementary roles of the measures chosen for this study are probably best explained by using Figure 2a (adapted from [52]). The Venn diagram presentation, also referred to as info-diagrams [53,54], depicts a time series divided into past and future. The dynamic of both are measured in terms of their entropies H, calculated by Shannon’s famous formula $H\left(X\right)=-\sum p\left(x\right)*log_{2}p\left(x\right)$ [42]. In our case, set X consists of an alphabet with either 20 or 200 different symbols, each binning bid-ask spreads of different sizes over period of two months. Entropy measures the uncertainty among the different events in terms of the uniformity of their distribution. It is at its maximum if all possible events are equally likely (maximum uncertainty), and zero if only one of all possible events occurs (total predictability).

In order to identify the required statistics, we use a sliding-window approach on the empirically recorded sequence and record the frequencies with which different combinations of subsequences occur (Figure 2b). We denote the length of the window with *L*. These are the fundamental structural units we work with for the purpose of prediction. We then quantify the amount of structure that is relevant for prediction (namely with the complementary measures *E* and *C*), and how much uncertainty remains, conditioned on the identified structure (namely with *h*) (Figure 2a).

#### 4.1.1. Complexity

*Predictable Information.* What we call ‘predictable information’, *E*, is also known as excess entropy in the literature [55] or ‘effective measure complexity’ [50,56], hence our symbol *E*. It is also known as ‘stored information’ [57], or ‘predictive information’ [58] (with slight differences regarding their asymptotic or time bound outlooks). It is defined as the mutual information [42] between the past and the future. From a perspective of information-theoretic channels, it is the amount of information that the past communicates to the future. It is the intersection between the entropy of the past and the entropy of the future (see Figure 2a). Scholars more familiar with linear statistics can see an analogy of this measure with what is known as autocorrelation. We call it ‘predictable information’, because it is the amount of structure from the past that is useful to predict the future. The more subsequences from the past are useful to predict the future, the higher the predictable information.

*Predictive Complexity.* What we call ‘predictive complexity’, *C*, is the statistical complexity of the time series, as captured by its optimal hidden-Markov model presentation, aka its ϵ-machine (say ‘epsilon machine’) [49]. *C* cannot be smaller than *E* (see Figure 2a), as it quantifies the amount of information required to predict the structure contained in *E* [59]. In other words, *C* “captures the minimal amount of information that a process must store to communicate all [‘predictable information’, *E*]… from the past to the future” [52]. *C* is a practical approximation of the Kolmogorov complexity of a dynamic, in a sense that it measures the size of the smallest model with maximal predictive power for the time series [60]. It is derived from the smallest size, optimally predictive, unifilar (deterministic) hidden Markov model of the dynamic, called an ‘epsilon-machine’ (see Figure 9, at the end of the article, for a visualization) [59,61]. *C* quantifies the size of this statistical representation of the model in terms of the entropy of its states (measured in bits). The amounts of bits of the predictive complexity *C* of a dynamic measure the minimum amount of information required to predict the future of that dynamic from its past optimally. The more bits needed to make optimal predictions, the more complex the process. *C* is zero for both deterministic and random processes, and it is maximal for stochastic processes with large memory effects.

#### 4.1.2. Remaining Uncertainty

Predictions are rarely perfect. Information-theoretic dynamical systems theory quantifies the remaining amount of uncertainty about the future given the past with the conditional entropy H(future|past). This term quantifies the remaining noise or measurement error. It measures how many bits of uncertainty are still left about the future when knowing the past (including everything the past can tell us about the future) (see Figure 2a). It is often expressed as a per symbol rate, *h*, which scales with the chosen window length *L*. The resulting entropy rate measures the uncertainty of the next turn conditioned on the previous turns [44]. In short, the entropy rate “gives the source’s intrinsic randomness, discounting correlations that occur over any length scale” [60]

An intuitive implication of the remaining uncertainty is captured by Fano’s inequality, which provides an upper limit to the probability of error of any prediction in terms of the process’s entropy rate: the larger the remaining uncertainty *h*, the larger the probability of prediction error, and the less accurate the predictions [42,62]. In this sense, one can think of the entropy rate *h* as a limit to predictability. The more remaining uncertainty, the larger must the prediction error, and the more unpredictable the future.

#### 4.1.3. Derivation of Measures

Summing up, the predictive complexity *C* measures the amount of information required to predict *E* bits of predictable information in the dynamic, while *h* quantifies the remaining uncertainty per upcoming symbol that cannot be predicted from the captured complexities alone. In other words, the predictive complexity *C* is the size of the optimal machinery required to predict all predictable information *E* about the future that is obtainable from the past, and *h* is the remaining uncertainty.

In practice, the nonlinear benefits of information-theoretic measures come with the notorious disadvantage of converging very slowly, which might lead to inconsistencies even with our ‘big data’. As with all statistics, they are also sensitive to different measurement decisions, but come with the additional disadvantage that best measurement practices are less agreed upon as in traditional linear statistics. We therefore ran a series of complementary tests, using two different ways to derive the measures. Our main presentation uses the Python implementation [63] of the Causal State Splitting Reconstruction (CSSR) algorithm for inferring epsilon-machines [64]. We denote measures derived by this method with subscript [__eM_] (using the spectral decomposition [65]). We confirm the results for *E* and *h* with estimates derived with the Python package dit [66], which uses another derivation method, more directly frequency-based, and denote them with subscript [__fq_] [48]. We also used cross-validation and other comparisons to test out different sampling-window length, being cautious with overfitting. Eventually, simplifying computational demands, we used window length of *L* = 2 for the method [__eM_], and *L* = 4 for the less computationally demanding derivation method [__fq_].

The main script for deriving all three measures, *C*, *E*, and *h*, can be found online in Python (https://github.com/ddarmon/transCSSR). A script to run it automatically over many input sequences from a csv file was added (https://github.com/3tz/transCSSR). R code for deriving *E* and *h* can be found here (https://github.com/martinhilbert/RURO-measures-and-plots). Calculating all of our required measures from our almost one billion data points took about a total of three weeks of 24/7 computing power on a 32 RAM desk PC (one week for running data wrangling tasks, two times times one week to derive measures.

We find strong correlations between the results from both derivation methods. Pearson correlation coefficients r between methods [_eM] and [_fq], for h_20 = 0.89; h_200 = 0.86; E_20 = 0.94; E_200 = 0.77. In this sense, we grew confident that our general conclusions are robust with regard to different estimation methods of these measures. We still present all results individually in the Supporting Information SI).

### 4.2. Independent Variables

#### 4.2.1. Algorithmic Trading

**Figure 3:**
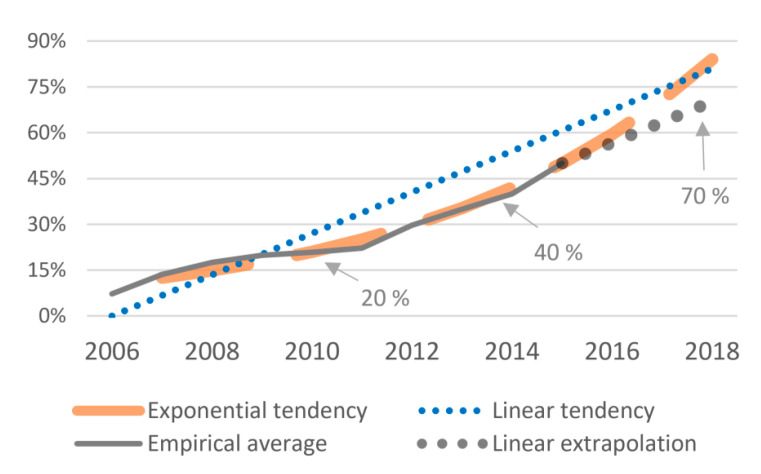
Estimated tendencies of the growing market participation of algorithmic trading.

The biggest empirical challenge was to obtain data of the evolution of the level of algorithmic trading. We did an extensive search for available estimates (see Figure 1) and created what we suggest to be a reasonable estimate for the general tendency of the growth of algorithmic involvement in foreign exchange markets (based on the numbers reported by [9,11,12,13,15,18,19,20]). The grey line in Figure 3 is the same as the average grey line in Figure 1b. We add both a linear trend line (AT_lin_) and an exponential trend line (AT_exp_), and extrapolate the empirical average with a linear tendency for 2016–2017 (which we denote with AT_emp_). This gives us three general estimates for the increase of the level of algorithmic trading in FX markets

In the following, we will mainly report the results when working with the empirical average and its linear extrapolation (AT_emp_), but we also test for the linear and exponential tendencies. It turns out that our results are very robust regardless of the choice among those three options. In other words, the resulting effects are very similar with any tendency that has monotonically increased over the decade, growing from less than 15% before 2007 up to some 70% in 2018. Since we are not aware of any other relevant variable that has shown such substantial and monotonic growth during this period, we increasingly became less worried about the precise shape of the empirical rise of algorithmic trading. This makes us quite confident to suggest that the role of any such monotonically increasing tendency represents some aspect of the growing involvement of algorithmic trading. At the least, it allows us to draw comparative conclusions in the context of our control variables (see below), none of which shows such monotonically increasing tendency. In other words, we detect effects of something other than the usual economic indicators that has substantially and monotonically increased over the decade. For lack of any other reasonable candidate for this variable, we argue that this effect stems from the increasing involvement of algorithmic trading.

#### 4.2.2. Control Variables

This being said, we need to control for several potential confounders that could have affected the increase in trading dynamics. The economic indicators used to forecast foreign exchange rate are usually taken to be the same traditional indicators customary used to determine the overall health of an economy. In fact, the intricate relation between them and the exchange rates makes the exchange rate itself one of the top indicators to identify the health of a country’s economy. According to Beers [67], the most common indicators to determine exchange rates are Gross Domestic Product (GDP), price index, interest rates and employment data. We obtained these four indicators for each involved economy from the databases of [68], namely for Australia, Euro-Zone, Japan, Great Brittan, and the United States (monthly data for inflation rate, interest rate, and unemployment rate, and quarterly data for the GDP growth rate (which we linearly interpolated to obtain our bi-monthly estimates). In order to reduce the number of variables (given our relatively small sample size), we took the difference between each currency pair and so obtained one number per currency pair.

Additionally, researchers often include a lagged value of the dependent variable as an independent variable in what is known as dynamic panel data models. In our case, it is to be expected that one of the best predictors of the dynamical signature in the next bi-monthly period *t*, is what happened at the previous period *t* − 1. Therefore, we also use the lagged dependent variable as another independent control variables. Since there is discussion about the practice of including lagged value dependent variable in panel data models [69], we also ran the exercise without it, and present the result in the Supporting Information SI.2. The results without lagged term reinforce the conclusions drawn in this study about the role of algorithmic trading.

#### 4.2.3. Variable Versions

Summing up, we are left with several versions of different variables that allows us to run a mutually re-conforming set of models. In general, we test for one of five dependent response variables at a time, using six independent predictor variables in each test.

- Three dependent variables (DVs). We derive different versions of our three summary measures, namely from ϵ-machines (epsilon-machine, _eM_) and from frequency counts (_fq_), calculated on basis of coarse-grained 20 bins (__20_) and more fine-grained 200 bins (__200_).
  ○ predictable information (*E*_eM_20_, *E*_fq_20_ and *E*_eM_200_, *E*_fq_200_)
  ○ predictive complexity (*C*_eM_20_ and *C*_eM_200_)
  ○ remaining uncertainty (*h*_eM_20_, *h*_fq_20_ and *h*_eM_200_, *h*_fq_200_)
- Six independent variables (IVs). Our main variable of algorithmic trading is estimated in three different ways (see Figure 3).
  ○ algorithmic trading
    ▪ empirical with linear extrapolation (*AT_emp_*)
    ▪ linear tendency (AT_lin_)
    ▪ exponential tendency (AT_exp_)
  ○ lagged dependent variable (dep_t−1_)
  ○ GDP growth rate (GDPr)
  ○ inflation rate (infl)
  ○ interest rate (intr)
  ○ unemployment rate (unpl)

We standardize each of the eight currency sequences (each 66 bi-monthly periods long) by subtracting its mean and divide by its standard deviation, joining all resulting 520 consecutive datapoints into one single database with (8 currencies * 6 bi-monthly periods per year * 11 years − 8 first measurements of each currency to obtain lagged value t − 1).

## 5. Results

We test our hypotheses with multiple linear regression of our six IVs to predict one of our DVs at a time. We ran diagnostic analysis on the residuals and feel comfortable with the multiple linear regression with normal noise model (see Supplementary Information SI.1).

It is essential to point out that we are running correlations here, which do not necessarily imply identification or establish causality. The establishment of causality would require either a theoretical economic model to explain the effects of algorithmification on trading dynamics [70,71,72], and we are unaware of any, or a source of exogenous variation on algorithmic trading [73,74], to reduce the likelihood of other confounding variables. An instrumental variable seems challenging to establish since our potential cause is only an approximation (see Figure 3). Therefore, we cannot rule out any other potential confounder (additional to the ones we control for). Any suggested identification of directionality within our correlations is also not derived from an empirical, but rather from a logical argument: it is simply less likely that a changing dynamic in bid-asked spreads (somehow caused by other factors) was the main initial driver for the introduction of algorithmic trading. Digital algorithms are a general-purpose technology that came into existence given the provision of the required technological infrastructure and low cost data, which made it possible to execute algorithmic trades. It is therefore more likely that this broad technological change affected bid-ask spreads, and not that changes in bid-ask spread dynamics in foreign exchange markets were the main driver of this omnipresent technological revolution. There might be positive feedback that involves a competitive race between increased market pressure and increased use of algorithms, but rather that that some exogenous effect on changed bid-ask spreads kick-started this race, and more plausible that it is largely driven by the exogenous introduction of cumulative algorithmic trading itself. This argument favor the claim that within this correlation, algorithmic trading affects trading dynamics, not vice versa. Of course, this does not eliminate the possibility of exogenous confounding variables.

### 5.1. Increasing Complexity (H1)

**Figure 4:**
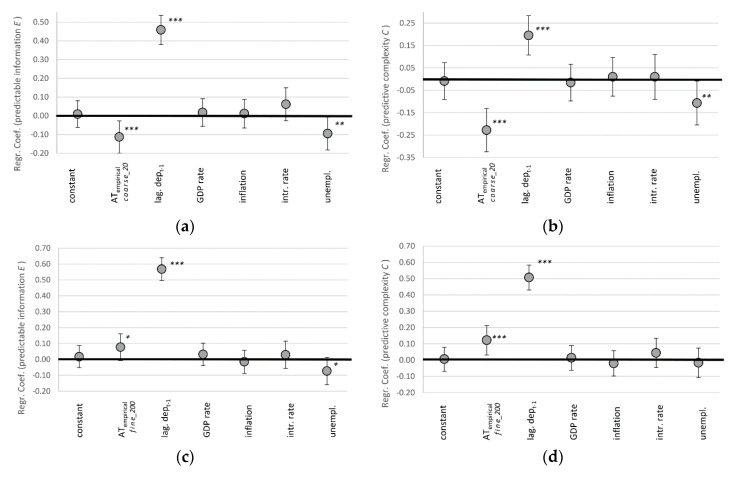
Regression coefficients for bi-monthly changes in complexity in form of predictable information *E*_eM_ (**a**,**c**) and predictive complexity *C* (**b**,**d**), where algorithmic trading is measured in 20 coarse-grained bins (**a**,**b**) and 200 fine-grained bins (**c**,**d**), indicating 95% confidence intervals with error bars. *** *p* < 0.01, ** *p* < 0.05, * *p* < 0.1 (N = 520).

Our two complementary measures for complexity are predictable information (also known as ‘effective measure complexity’ [50,56]), and predictive complexity (also known as ‘statistical complexity’ [49]). We test the resulting dynamical signatures from a rather coarse-grained perspective on basis of 20 bins (__20_) and more fine-grained 200 bins (__200_). For reasons of didactic presentations, Figure 4 showcases the results only for the [*_*_eM_] derivation method and the empirical AT_emp_ estimate. We provide the full tables for all 18 models in Supporting Information SI.3. The version visualized in Figure 4 is among the models where AT has the least influence, and is therefore a rather conservative estimate of our broader results.

The lagged variable of AT autocorrelation is the strongest predictor of the changing complexity of trading dynamics. Independent from any path dependency, our estimate for algorithmic trading is the only other consistently significant predictor. It also has the largest effect size among our tested variables. It is interesting to note that for the case of our coarse-grained perspective of bid-ask spreads (20 bins), algorithmic trading is associated with less complexity (Figure 4a,b). Only accounting for trades at a more fine-grained level (200 bins), one more digit behind the comma, it turns out that higher levels of algorithmic trading are positively correlated with complexity (Figure 4c,d).

We therefore reject our hypothesis that the involvement of algorithms correlates positively with more complex trading dynamics from the coarse-grained perspective. However, going one digit deeper, we cannot reject the hypothesis. Figuratively speaking, when trading on the levels of dimes (larger margins), things got less complex; but when trading on the more detailed levels of pennies (smaller margins), things got more complex over the last decade.

### 5.2. Decreasing Uncertainty (H2)

**Figure 5:**
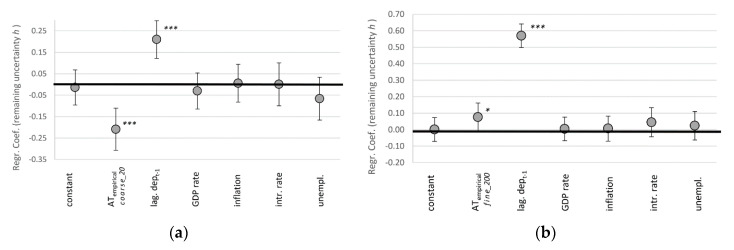
Regression coefficients for bi-monthly changes in remaining uncertainty in form of entropy rate *h*_eM_, where algorithmic trading is measured in 20 coarse-grained bins (**a**) and 200 fine-grained bins (**b**), indicating 95% confidence intervals with error bars. *** *p* < 0.01, ** *p* < 0.05, * *p* < 0.1 (N = 520).

Our measures for remaining uncertainty is the dynamic’s entropy rate *h* (more entropy, more uncertainty), which we also measure based on coarse-grained 20 bins-perspective, and the fine-grained 200 bins-perspective. Again, for reasons of argument flow, Figure 5 only visualizes the results for the [*_*_eM_] derivation method and the empirical AT_emp_ estimate (for the full array of all 12 models, see Supporting Information SI.4). All models support the conclusions presented here.

For the remaining uncertainty, we find similar results as we did for complexity. The coarse-grained perspective of larger margins is in line with our hypothesis: uncertainty is negatively correlated with AT (Figure 5a). There is less surprise in the dynamics of bid-ask spreads as the prominence of algorithmic trading increased. The predictive power of AT on the bid-ask spread dynamic is as large as the spread’s autocorrelation (Figure 5a). However, from the fine-grained perspective we have to reject our hypothesis H2: barring path dependencies, increasing algorithmic trading is the best predictor of increased uncertainty in foreign exchange markets. This result is confirmed and even stronger and more significant for all other models identified in SI.4.

### 5.3. Robustness of Results for Complexity and Uncertainty (H1 & H2)

It is insightful to join the results from the foregoing hypotheses H1 and H2 into a single analysis and look at our complementary measures from a slightly different perspective. For this, we ran different ANCOVAs (analysis of covariance), which naturally leads to similar results than multiple regression, given the close relation between both methods. The analysis loses some power, since we dichotomize the scalar variable of algorithmic trading into a ternary fix factor that tracks the thirds of bi-monthly periods (with least algorithmic trading, the mid-level, and the most algorithmic involvement, likewise oldest and most recent data, given monotonicity of our AT variable in time). This perspective has the benefit that it lends itself to memorable visualizations in form of so-called “complexity-entropy diagrams”, which have been widely used in complex systems literature to show the “diversity of natural information processing” in dynamical systems [75]. Colloquially, this form of presentation is sometimes referred to as ‘complextropy plots’.

**Figure 6:**
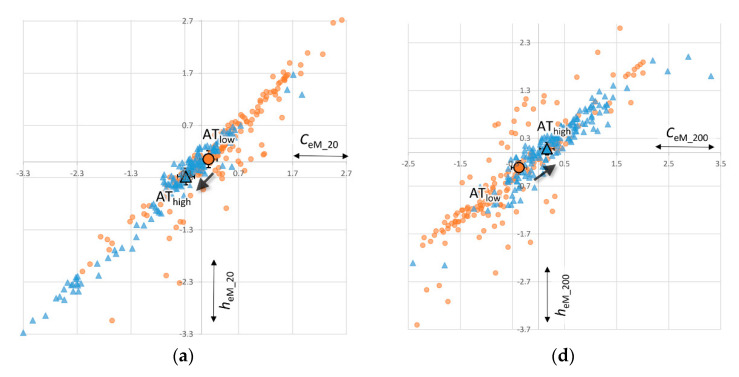

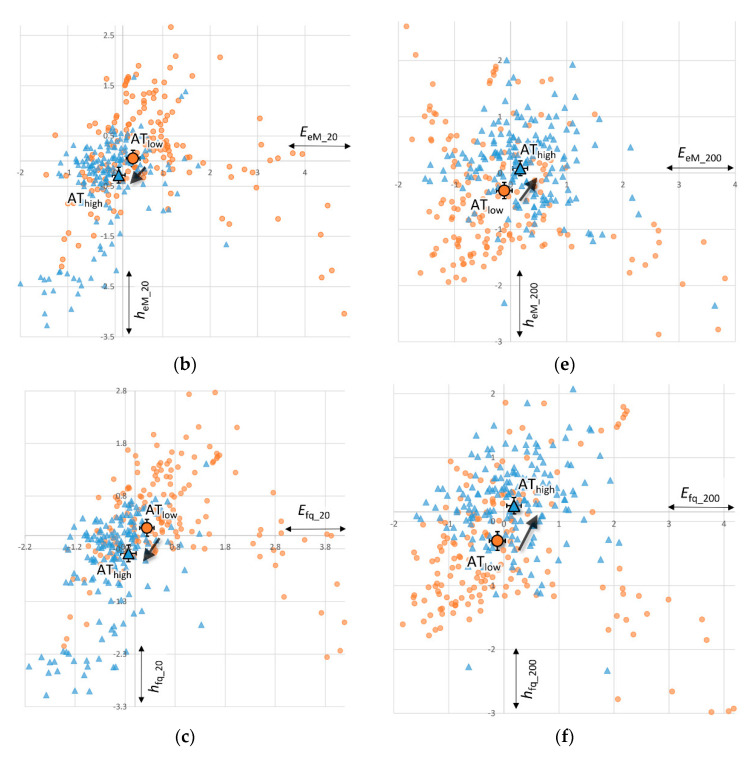
Estimated marginal means (large dots) from ANVOCA (95% confidence error bars), with thirds of AT_emp_ as fixed factor, controlling for the covariates dep_t−1_, GDPr, infl, intr, unpl; orange circles: third with lowest algorithmic trading (AT_low_); blue triangles: third with highest algorithmic trading (AT_high_); (**a**–**c**) for coarse-grained perspective of 20 bins, (**d**–**f**) for fine-grained perspective of 200 bins. Note: in contrary to means, scatter data points are not corrected for control variables and some outlier are cut off by presentation.

Figure 6a–c visualize how both complexity and uncertainty have been reduced from the perspective of larger coarse-grained margins. Trading on the nth digit behind the comma (e.g., the 4th digit behind the dollar), dynamics became less complex, and more predictable. On the contrary, Figure 6d–f show that trading on the nth + 1 digit behind the comma (e.g., the 5th digit behind the dollar), dynamics became both more complex and more uncertain. The tendency of this result is robust among our different estimation methods and models. Figure 6b,c,e,f visualize the similarity and slight differences between our [*_*_eM_] and [*_*_fq_] derivation methods. The confidence intervals (see error bars at estimated marginal means) clearly do not overlap, which shows that the dynamics have changed significantly in the indicated directions.

## 6. Discussion and Interpretation

Not surprisingly, in line with the well-known folk wisdom about weather forecasting, the best predictor of tomorrow’s trading dynamics are today’s. Predictable and simplistic periods are likely to be followed by another two months of the same. Likewise for turbulently unpredictable and complex dynamics. Independent from this local path dependency, our estimate for algorithmic trading is a better predictor of changing trade dynamics than what traditionally are considered to be the best predictors of trade (namely GDP growth rate, inflation, interest rate, and unemployment rate [67]). Our results are fairly consistent: one standard deviation increase in algorithmic trading reliably predicts a 0.1 standard deviation increase in the associated information-theoretic measure of the ongoing dynamics

Some of our estimates are borderline significant. Surveying the nature of the residuals in our models and the coherent results we obtained from our different models, we expect that the statistical significance would increase with larger sample sizes that allow for more precise estimation of the coefficients (we have 520 data points and 6 independent variables).

### 6.1. There’s Plenty of Room at the Bottom

**Figure 7:**
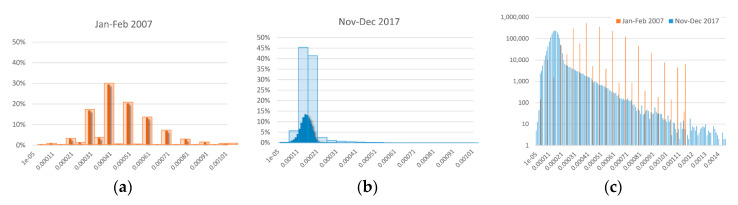
Frequency distribution of bid-ask spreads EUR/AUD (**a**) Jan.–Feb. 2007, unbinned and in 20 bins; (**b**) Nov.–Dec. 2017, unbinned and in 20 bins; (**c**) unbinned both 2007 and 2017, logarithmic scale.

Our opposing results for the more coarse-grained and fine-grained perspective suggests that a major driver for the results stems from changes in how pip pricing are handled (point in percentage, in our case of bid-ask spreads). Figure 7 visualizes this interpretation in the case of the EUR/AUD bid-ask spread. In 2007 (Figure 7a), traders mainly traded on round numbers, even so it was possible to trade on a finer decimal level on the platform (our data shows there are occasional trades right next to round numbers, but those trades were not the norm). As early as the 1960 [76], it was known that “stock market decision makers place their limit and stop orders at numbers with which they are accustomed to deal” [77], which are mainly even and round number. “The phenomena is remarkably persistent through time and across stocks. Clustering distributions from the mid-nineteenth century appear no different from those observed in the late twentieth century” [78]. After FX exchanges fine-grained trade from pip to decimal pip, human traders still placed at or halfway between the old allowed prices, while trading algorithms “have no reason to not take advantage of decimal pip pricing” [79].

There are a number of proposals to explain the clustering property of human traders around coarse-grained round numbers [80]. They reach from missing information to make more detailed decisions [81], over a natural attraction to even numbers [82], to an artificial manipulation of prices on behalf of the traders [83,84]. One hypothesis holds that traders coordinate to restrict themselves to a smaller set of prices in order to reduce negotiation costs either implicitly or explicitly [78]. We asked a leading FX trader of a major Swiss bank for his interpretation, and he asserted that—given the amounts usually traded in FX market accounts—it is not worth the time of a human trader to pick up the phone and negotiate a trade on the fifth digit behind the dollar. There was, and still is money to be made on the fourth digit behind the dollar. According to him, the fifth digit is beyond human transaction costs, and in the realm of algorithmic efficiency.

Lack of and inability to process detailed information and related transition costs would suggest that algorithms that are able to compute faster than humans would not find any need to cluster around round numbers. Schmidt ([11], p. 4) found that “about 80% of MT [manual trading] were using decimal pricing in not more than 20% of all their orders. On the other hand, about 55% of AI [automated interface trading] were using decimal pricing in more than 80% of their orders.” Even more so, there is compelling evidence that algorithmic traders identified the human bias toward round numbers and took advantage of it, using sub-penny strategies to exploit the smaller tick size to obtain a margin [6,80]. Once a human trader places an order at a round number with last digit zero, on the ask side, algorithmic “queue jumping is done by submitting an order with last digit 9 and the buy side by an order with last digit 1… Automated traders queue-jump first the manual trader quote then leap frog each other as they compete for top of the book” [79]. Algorithms advanced into the more detailed aspect of reality where humans did not [85]. Summing up, a study group of 14 central banks comes to the conclusion that “while algorithms can relatively easily handle the extra digit, human traders find it more difficult to adapt” [19].

The result of this is shown in Figure 7b,c. For one, the average bid-ask spread was reduced (see a more in-depth analysis of this in SI.7). Additionally, no space is left between neighboring spreads. Using the same scale to compare both cases, rather wide bins are enough to adequately capture the round-number trading of 2007. During Jan.–Feb. 2007, we only find 24 different bid-ask spreads. Most of them at round numbers. Of these, the largest bin captured 30% of the possibilities (Figure 7a), which leads to a rather uniform distribution. Adopting the same coarse-grained view a decade later leads to a rather skewed distribution, where one single bin captures more than 45% of all possible spreads (Figure 7b). From the coarse-grained perspective that was adequate in 2007, uncertainty about the next bid-ask spread is reduced in 2017: the next trade will almost certainly occur in one of the lower spread bins. This is in agreement with the results from [32], who found decreasing predictive complexity of the S&P500, using a very coarse-grained binary binning. However, we find that within those bins, a more fine-grained level of uncertainty has opened up. We find 145 different bid-ask spreads in Nov.–Dec. 2017. From this fine-grained perspective, there is more uncertainty about the next bid-ask spread: there are more possible spreads. The same holds for all currencies (see Supporting Information SI.5).

In other words, algorithmic trading contributed to decrease the uncertainty relative to the perspective human traders were accustomed to in the mid-2000s, while they introduced a new level of uncertainty on a more fine-grained level. Algorithms automate the world as it is, eliminating uncertainty on that level, while at the same time, deepening our conception of reality, digging one level deeper. The more detailed perception produces more possibilities than we were accustomed to before, which leads to our detected result of increased uncertainty. This reaffirms Richard Feynman’s [86] famous insight that there is plenty of room at the bottom: in our case, with the help of algorithms, at the fine-grained bottom of trading markets.

### 6.2. Digging Deeper: The Chain Rule of Entropy

As a methodological aspect of our final discussion, it is interesting to point out that one can conveniently calculate the magnitude of this fine-graining uncertainty effect with the chain rule of entropy [42]. The chain rule adds lower-level fine-grained entropy to higher level coarse-grained entropy. In alignment with the presentation in Figure 7a,b, we first calculate the entropy of this more transparent binning, and then add the remaining uncertainty within each bin.

(1a) Chain rule of entropy: H(X,Y)=H(X)+H(Y|X)

(1b) H(20bins,200bins)=H(20bins)+H(200bins|20bins)

Note that our multilevel case is a special nested case of the chain rule. Turning the conditioning variable around, we can always reconstruct the low resolution uncertainty by aggregating the details: H(coarse | fine) = 0. There is no remaining uncertainty about the 20 bins level when we know which of the 200 bins is being traded. H(20bins,200bins) = H(200bins) + H(20bins|200bins). This can, but need not hold the other way around.

Applying Equation (1b) to the case of Figure 7a, it shows that the more fine-grained uncertainty did not exist in 2007. The conditional entropy is 0: knowing the coarse-grained perspective is enough to know everything about the fine-grained perspective, since there was no fine-grained trading (see Equation (2a)). However, in the case of Figure 7b, the more detailed perspective doubled the involved amount of uncertainty, adding an additional 2.18 bits of conditional entropy Equation (2b).

(2a) EUR/AUD Jan.–Feb. 2007: H(20bins,200bins)=2.86 bits=2.86 bits+0

(2b) EUR/AUD Nov.–Dec. 2017: H(20bins,200bins)=3.90 bits=1.72 bits+2.18 bits

**Figure 8:**
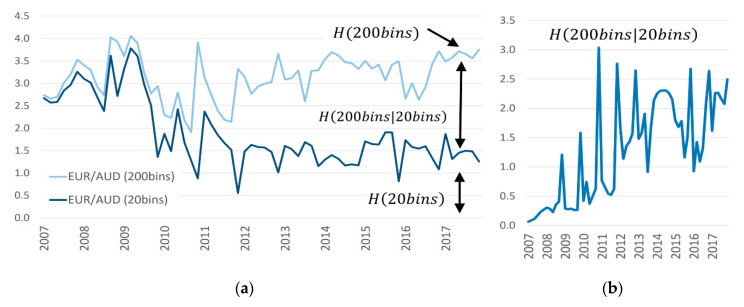
Chain rule of entropy applied to EUR/AUD bid-ask spreads, with 20 and 200 bins. (**a**) visualizes the diverging tendency over time; (**b**) isolates the size of this area.

Figure 8 visualizes the diverging tendency over time. The area between both levels of uncertainty is the conditional entropy (conditioned on the more coarse-grained resolution). Figure 8b isolates the size of this area: H(200bins|20bins). It shows that there was a notable change around 2011, which is the time when it is estimated that algorithmic trading increased its market penetration (see Figure 1 and Figure 3). There was no regulatory change during this time, just behavioral change, as algorithmic trading became mainstream in FX trading. The same hold for the other currency pairs (see Supporting Information SI.6).

### 6.3. The More You Know, the More Uncertain You Get

Another finding worthy of more discussion is the positive correlation between predictive complexity *C* and remaining uncertainty *h*, which can be seen in Figure 6a,b. There is no mathematical necessity for both to be associated deterministically, but in practice, both are often associated, given the nature of their origin. Their theoretical relation gives an intuitive way to interpret our results of both increasing simultaneously.

**Figure 9:**
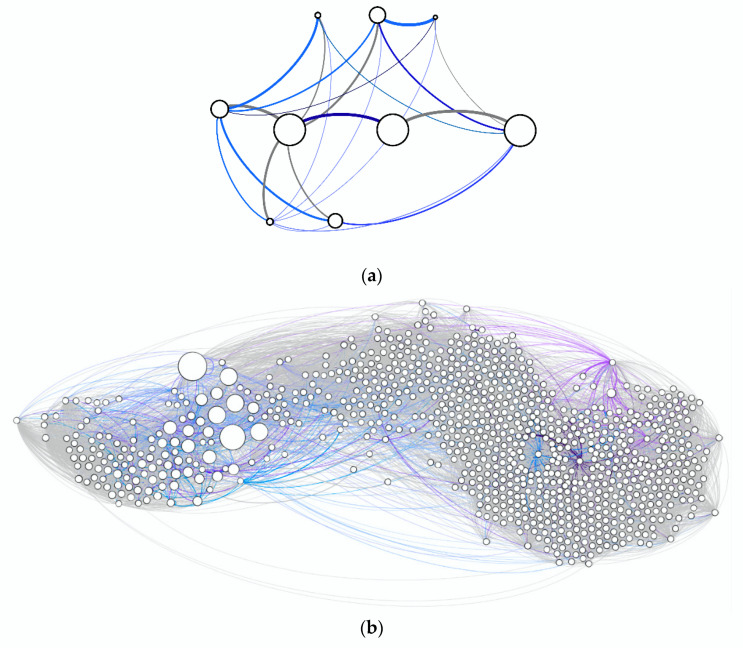
Visualization of ϵ-machines (aka predictive state machines) derived for (**a**) AUD/JPY Mar.–Apr. 2009, 200 bins; (**b**) EUR/AUD Jan.–Feb. 2017, 200 bins. The size of a node represents the steady state probability of the corresponding state. Clockwise curve indicates transition directionality. Transition color corresponds to symbols in the alphabet in the sequence.

Three decades of research on what is known as ‘computational mechanics’ [49,52,59,87,88] have shown that all three of the commonly used information measures *E*, *h*, an *C* can be derived from so-called ϵ-machines (aka predictive state machines). An ϵ-machine is the smallest size, optimally predictive, deterministic hidden Markov model for a given stochastic process (examples shown in Figure 9). The hidden states are created by joining those histories that predict the same future distribution of possible events. “At root, extracting a process’s representation is a very straightforward notion: do not distinguish histories that make the same predictions. Once we group histories in this way, the groups themselves capture the relevant information for predicting the future” [60]. The predictive complexity *C* then quantifies the size of this statistical representation of the model in terms of the entropy of the stationary distribution of the (hidden) states. The justification for summarizing complexity with entropy lies in Shannon’s famous source-coding theorem [44], which holds that “entropy is the minimum descriptive complexity of a random variable” [42].

Entropy increases when the underlying random variable is both more uniform and has more realizations (aka a larger alphabet), or, in this case, with more states. The more states, the more uncertainty about which state predicts the next future event, and therefore the larger the descriptive complexity of the hidden Markov model (i.e., larger predictive complexity *C*). Once the ϵ-machine is derived, a convenient way to obtain the entropy rate of the underlying dynamic turns out to be from the transition probabilities between states. It is a well-known result from stochastic process theory that “the entropy rate, which is the average transition entropy, depends only on the entropy of the stationary distribution and the total number of edges” [42]. The more states, the more possible transitions, the more potential uncertainty about where the model’s dynamic moves next. The probabilistic nature of the transitions gives the dynamic its intrinsic randomness, its remaining uncertainty *h*.

On average, our derived ϵ-machines have 203 predictive states (total of 1,188 machines), with a minimum of nine states (AUD/JPY Mar.–Apr. 2009) and a maximum of 894 states (EUR/AUD Jan.–Feb. 2017) (see Figure 9) (Our 528 ϵ-machines based on 200 bins of the bid-ask spread variable have on average 264 predictive states. The 528 ϵ-machines based on 20 bins of the bid-ask spread variable have on average 141 states, with a minimum of 11 states (EUR/USD, Nov.–Dec. 2015), and a maximum of 604 states (AUD/JPY, Jul.–Aug. 2017)). The specification of the nodes requires more than 3 bits more for the larger machine (*C* = 5.79 bits versus *C* = 2.66 bits, being the entropy of the steady state probability of the states). The intrinsic uncertainty of the process is 2 bits larger for the larger machine (*h* = 0.59 bits versus *h* = 2.59 bits, being the entropy rate). The larger machine requires both more information to specify the process’s state, and to determine where it goes next.

It is important to repeat that there is no mathematical necessity for *C* and *h* to correlate in a specific way. In theory, there can be models with more uniformly distributed predictive states (larger *C*), but with deterministically determined transition probabilities between them (smaller *h*). However, these would need to be designed in a very specific way. In empirical reality, we usually find cases like the ones shown in Figure 9. Predictive state machines provide a formal way to think about the old insight that the more you can know about a process, the less you can know about it: the more predictable structure is embedded in the dynamic of a complex process (quantified by its minimum description length *C*), the more possibilities emerge to transition among the parts, and therefore, the more uncertainty emerges (quantified by the limit of predictability *h*). This leads to a natural positive correlation between predictability and uncertainty in complex systems.

Note that this relation between states and transitions does not automatically apply to our other measure of complexity, namely the mutual information between the past and future, *E*, which is a more common measure of complexity than *C*, used in fields as diverse as physics [50,55,56,57], neuroscience [58] and consciousness [89,90]. Even so *E* sets a lower bound on *C* (see Figure 2), this bound is often loose, and *C* and *E* do often not correlate strongly with each other (see also Figure 6). The Pearson correlation coefficients between *C* and *E* in our data for 20 bins is 0.22; for 200 bins it is 0.52.

## 7. Conclusions

We used well-established measures from dynamical systems theory to show that foreign exchange trading markets have become both more predictable and more uncertain at the same time. Among our variables, we identified algorithmic trading as the main predicter of this tendency (while we cannot rule out the existence of other potential confounders). While this seems contradictory at first sight, information theory allows us to identify the source of this result. The main source of confusion for our intuition is the fact that we instinctively assume that there is a finite amount of uncertainty, and that we will eventually saturate it all with our predictive power. The currently dominating machine learning paradigm feeds on aspects of the intuition that uncertainty can be replaced by predictable structures, if we only add more sophisticated “prediction machines” [1]. However, this intuition is wrong. To the contrary, usually, uncertainty tends to increase despite (and because) of the existence of more predictable structural patterns.

### 7.1. Infinitely More Levels of Uncertainty?

Figuratively speaking, instead of saturating some kind of metaphorical finite space of uncertainty with predictable structure, algorithmic complexity grows in an infinite space of uncertainty. Reality is not a finite space to be saturated with knowledge, but an infinite space, in which we ever advance to new levels. We have seen that algorithmic trading went hand in hand with reduced uncertainty and more predictable structure, especially on the coarse-grained perspective used a decade ago. However, new structure was built to dig deeper into a more fine-grained level of reality, where we more uncertainty emerged. We found plenty of room at a new bottom of trading dynamics. One might imagine that the increasing surface of the outline of the dynamics gets in touch with ever more possibilities as the structure of the dynamic grows more complex in an infinite space of uncertainty. The more complex structure we build into socio-economic dynamics, the more options open up for uncertainty (based on the newly built structure) and the less predictable tends to be the process overall. Algorithmification is associated with the creation of new structure in dynamical processes to an unprecedented degree. We found significant differences within only one decade. An often-neglected side effect is that algorithmification also correlates with an unprecedented amount of additional uncertainty. This contributes to profound discussions about the long-term future of trading, such as if this trend could continue during the decades to come.

Our explanation of our empirical results are rather obvious, in hindsight, which is interesting in light of the fact that we have not found literature that discusses these intuitive findings clearly. Our results naturally arose from applying the methodological tools from information theory and its extensions from theoretical computer science to dynamical systems. Coming from a perspective of information processing, we essentially assessed how trading markets compute. Specifically, we derived a version of finite-state machines, which are the main workhorse of theoretical computer science [91], and asked how to best compute the next bid-ask spread based on bi-monthly periods of the dynamic of a currency pair. “Computation theory defines the notion of a ‘machine’—a device for encoding the structures in discrete processes… Given this framework, one talks about the structure of the original process in terms of the complexity of the reconstructed machine” [61]. We found that the predictive state machines representing our market dynamics became both more complex, and contain more uncertainty. The reason, in information theoretic terms, was that the processes adopted a larger alphabet that describes more computational possibilities (the number of possible realizations increased). Going back to the origins of information theory, larger alphabets have more letters, implying more possibilities to construct words (predictable subsequences of the dynamic), and therefore both more possibilities to build new temporal structures and more uncertainty about the next letter to be communicated by the system. The language of computation and information processing seems to be a natural language to use when asking about the effects of algorithms to dynamical systems, aka “the complex multiplatform nature of modern computer trading environments” [4]. In this sense, our study also serves as a showcase for the utility of such measures and methods.

### 7.2. Limitations and Future Outlooks

We would also like to point to four important limitations of our study. First, our measures for the level of algorithmic trading (AT) is merely a proxy. We do not directly observe whether a particular order is generated by a computer algorithm, but take a macro perspective on market dynamics. We found several estimates to lead to very robust results (see Supporting Information), but our results would definitely benefit from complementary studies from the micro-perspective, with more concrete representations of algorithmic trading.

Second, our analysis does not establish causality, but merely a correlation. As for the directionality within the correlation, it is simply less likely that a changing dynamic in bid-asked spreads was the main initial driver for the introduction of algorithmic trading, than a vice-versa directionality. Still, this does not rule out that there might be an exogenous source acting as confounding variable, causing more complexity and/or uncertainty independent from AT. More detailed studies will need to look into this question and the question of causality in general.

Third, we model trading dynamics based on univariate time-series. Both of our measures of dynamical structure (namely *E* and *C*) capture exclusively forms of autoassociations within this univariate sequence. All causal effects from interactions with other variable are contained in the remaining uncertainty *h*. Recent advancements in information theory have provided tools to calculate multivariate nonlinear measures for dynamical systems, including transfer entropy [92], which is related to linear Granger causality [93,94], information decompositions [95,96], and ϵ-transducer [97]. For now, practical applications of these multivariate measures are limited to two or three variables, but they would allow to detect interdependencies among currency pairs. With sufficient data, and without computational constraints, one could even imagine estimating computational models for the entire stock market (of the sort of the finite state machines shown in Figure 9), including as many currencies and variables as are obtainable. The result would be a more accurate formal model of how the stock market computes, since it is to be expected that there are lots of interdependencies.

Fourth, in our study we neglected speed. Much of the literature on algorithmic trading has focused on the impressive nanosecond speed it introduced. Fiber-optic trading lines have been laid by rocksawing through mountains [40] and transatlantic cables have been built to allow trading algorithms to shave 5 milliseconds off stock trades [41]. In our analysis, we do not ask when trades happen, but what kind of trade will happen, independently from the period between trades. Speed and accuracy might be related. Some studies have suggested that “slowing down the agents increased the market’s overall ability to settle to a competitive equilibrium, and that slow-agent markets were more efficient” [98]. We would expect that the inclusion of the speed variable would provide a complementary outlook on the definition of complexity and uncertainty in trading dynamics.

## Supplementary Materials

The following are available online at https://www.mdpi.com/1099-4300/22/5/499/s1, Figure S1: Assumptions of Linear Regression, Figure S2: Comparison without Lagged Term, Figure S3: Histograms of Bid-Ask Spreads, Figure S4: Histograms standardized Bid-Ask Spreads, Figure S5: Conditional Entropy Plots, Figure S6: Decreasing Bid-Ask Spreads, Table S1: Full Array of Models for H1 coarse-grained, Table S2: Full Array of Models for H1 fine-grained, Table S3: Full Array of Models for H2 coarse-grained, Table S4: Full Array of Models for H2 fine-grained, Table S5: Decreasing Bid-Ask Spreads.
