# Supplementary material for: How Complexity and Uncertainty Grew with Algorithmic Trading
Source: Entropy (Basel). 2020 Apr 26;22(5):499. doi: 10.3390/e22050499 (PMC7516984; doi:10.3390/e22050499)
Supplement: Supplementary file 1 [file entropy-22-00499-s001.pdf]

Supporting Information

How Complexity and Uncertainty Grew with Algorithmic Trading

Martin Hilbert [1,*] and David Darmon [2]

[1] Communication, Computational Social Science; University of California, Davis, CA 95616, USA; hilbert@ucdavis.edu

[2] Department of Mathematics; Monmouth University, West Long Branch, NJ 07764, USA; ddarmon@monmouth.edu

SI.1. Assumptions of Linear Regression

We ran a series diagnostic analysis on the residuals of our multiple linear regressions and feel comfortable with the multiple linear regression with normal noise model. We found that the most worrisome violation stems from the normality of the residuals.

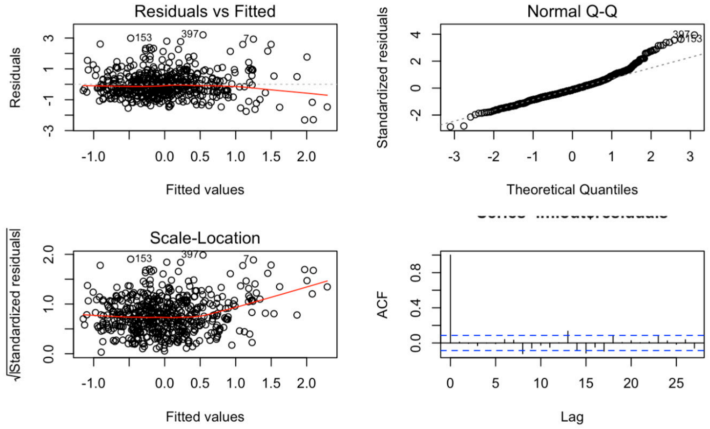

(a)

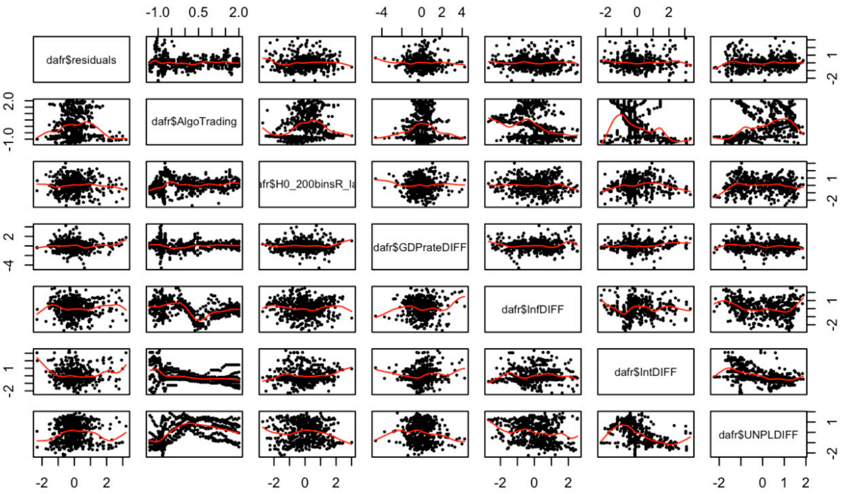

(b)

Figure S1. *Cont.*

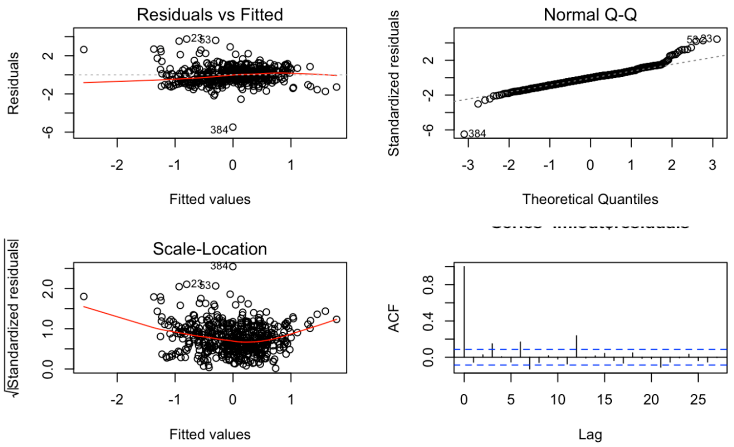

(c)

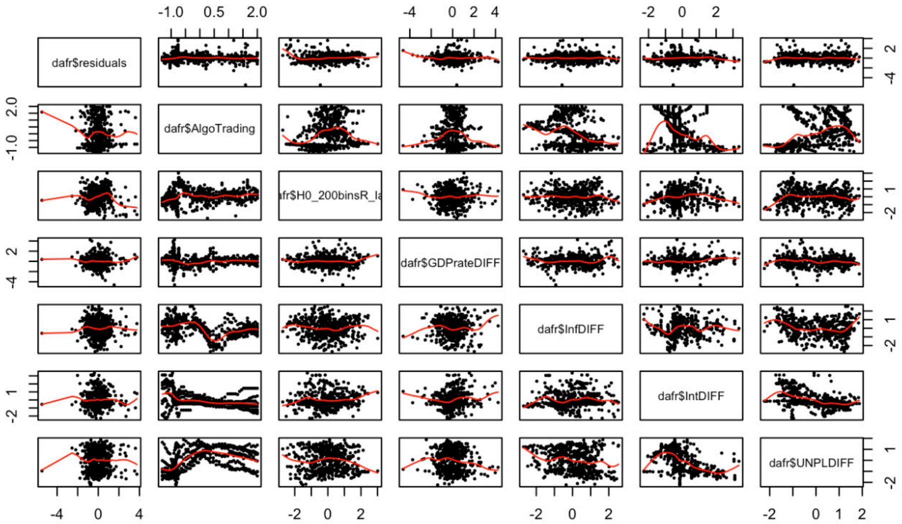

(d)

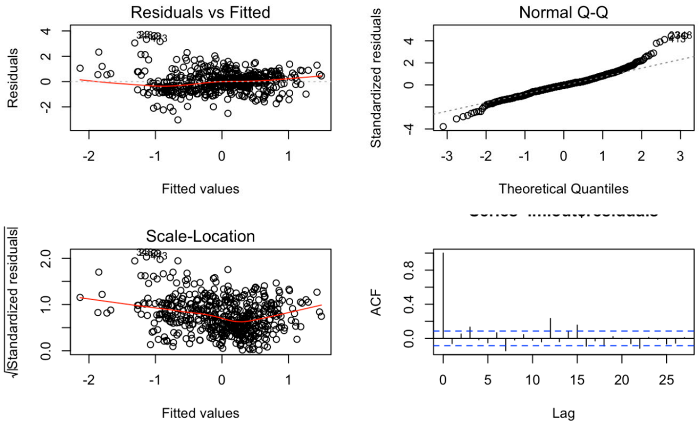

(e)

Figure S1. *Cont.*

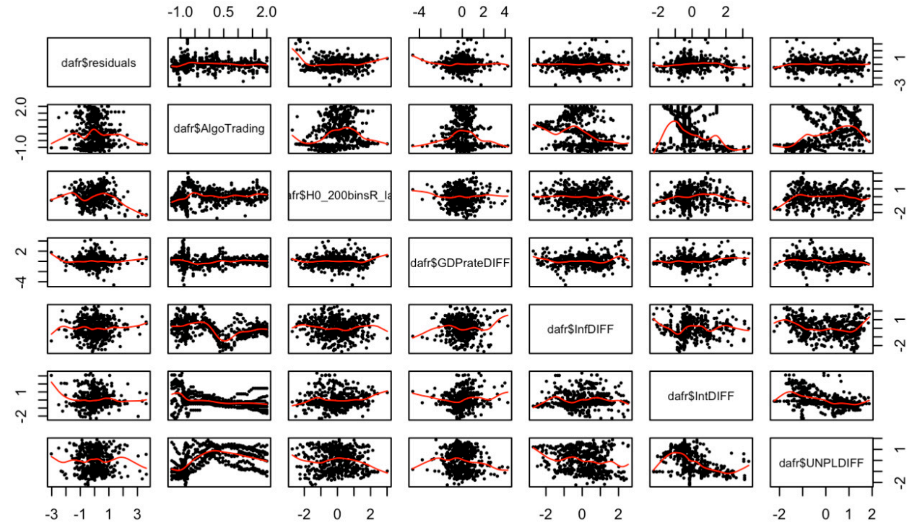

(f)

Figure SI.1. Residual diagnostics for normal noise model (a-b) E_{eM_200}, (c-d) C_{eM_200}, (e-f) h_{eM_200}.

Fortunately, this also tends to be the least concerning assumption, since a central limit theorem-based results makes the actual distribution of the residuals wash out with enough data. Given the nature of our data from eight different currency pairs, linked in time, any alternative bootstrapping procedure would have to be fairly intricately involved, which would reduce transparency and replicability. We feel that our normal noise assumptions are within what is generally accepted in the community.

SI.2. Comparison without Lagged Term

Social dynamics often contain an important path dependency. Therefore, researchers interested in other influences often include a lagged value of the dependent variable as an independent variable in what is known as dynamic panel data models. Our analysis confirms that the dynamics of the pervious bi-monthly period *t-1* is highly predictive of the next one *t*. In this sense, our results show the influence of the other tested variables independent from this effect of path dependency. However, there is an ongoing discussion about the practice of including lagged value dependent variable in panel data models [1]. Leaving out the lagged term leads to a lot of autocorrelation in the residuals of the regression, which would violate basic assumptions. Just to make sure, we also ran the exercise without it. The main conclusions drawn from this study are strongly reinforced when running the tests without the lagged term. The influence of algorithmic trading increases. For example, Figure SI.2 shows the case of measure E_{eM_200}. AT_{emp_200} is only weakly significant when considering lagged path dependency, but becomes significantly stronger without. The same is shown for measure h_{eM_200}, and in general applies to all tests we have seen. We present the version with lagged term in the main article, because it better corresponds to the basic assumptions of linear analysis, and because it presents the more conservative version of our results.

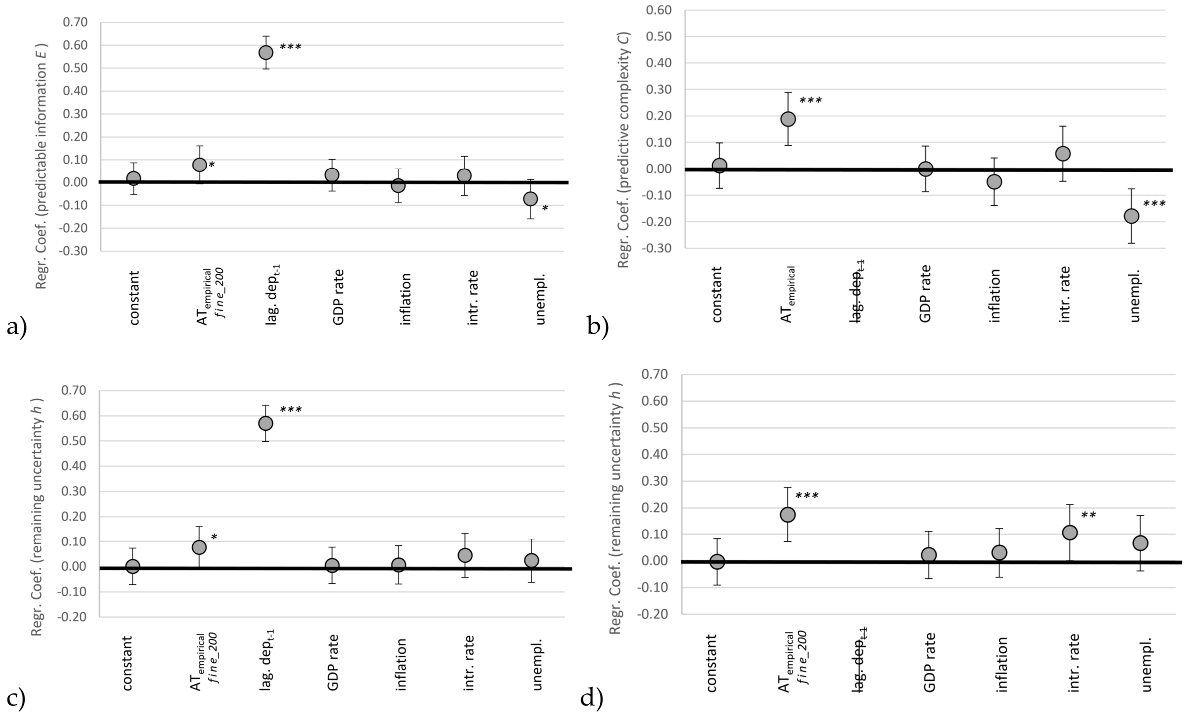

Figure SI.2 Regression coefficients for bi-monthly changes in dynamics measured in 200 fine-grained bins, indicating 95 % confidence intervals with error bars, (a) predictable information *E* with lagged term; (b) without lagged term; (c) remaining uncertainty *h* with lagged term; (b) without lagged term. *** p<0.01, ** p<0.05, * p<0.1 (N = 520).

SI.3. Full Array of Models for H1

As discussed in the main article, we also use three complementary estimates for the rise of algorithmic trading (AT), namely empirical, linear and exponential. Given that our information-theoretic dynamical-systems indicators are not as deeply established in the social sciences, and given that there are less agreed upon best practices in their estimation, we test their robustness by using two different methods to calculate them for each bi-monthly interval, namely ϵ-machines (epsilon machines, eM) [2] and frequency counts (fq) [3]. Note that predictive complexity is a measure of the associated ϵ-machine [4,5], and we only derive those once, with the Causal State Splitting Reconstruction (CSSR) algorithm.

It shows that our results are quite robust, independent from the derivation method, and independent from the estimate for the rise of algorithmic trading. In Tables SI.1 and SI.2, model (2) and model (7) are marked in bold since these are the ones presented in the main article. As can be seen, these are among the models where algorithmic trading has the least influence, and are therefore a rather conservative estimate of our broader results.

Table SI.1: Tests for bi-monthly changes in coarse-grained (20 bins based) complexity in form predictable information (E) and predictive complexity (C), measured according to frequency counts ($_{fq}$) and ϵ-machines ($_{eM}$); showing unstandardized beta coefficients, with standard errors in italic parentheses. *** p<0.01, ** p<0.05, * p<0.1 (N = 520).

Dep. Var.	(1) E_{fq_20}	(2) $\mathbf{E_{eM\_20}}$	(3) E_{fq_20}	(4) E_{eM_20}	(5) E_{fq_20}	(6) E_{eM_20}	(7) $\mathbf{C_{eM\_20}}$	(8) C_{eM_20}	(9) C_{eM_20}
constant	-0.003	**0.004**	-0.001	0.006	-0.003	0.004	**-0.009**	-0.008	-0.009
	(0.035)	***(0.036)***	*(0.035)*	*(0.036)*	*(0.035)*	*(0.036)*	***(0.041)***	*(0.041)*	*(0.041)*
AT_{emp}	-0.149***	**-0.118*** **					**-0.232*** **		
	(0.042)	***(0.043)***					***(0.049)***		
AT_{lin}			-0.199***	-0.173***				-0.231***	
			(0.045)	*(0.046)*				*(0.051)*	
AT_{exp}					-0.150***	-0.117***			-0.228***
					(0.042)	*(0.043)*			*(0.048)*
dep_{t-1}	0.412***	**0.454*** **	0.395***	0.438***	0.411***	0.454***	**0.193*** **	0.196***	0.195***
	(0.040)	***(0.039)***	*(0.041)*	*(0.039)*	*(0.040)*	*(0.039)*	***(0.044)***	*(0.044)*	*(0.044)*
GDPr	0.004	**0.013**	0.001	0.012	0.004	0.013	**-0.016**	-0.021	-0.016
	(0.036)	***(0.037)***	*(0.036)*	*(0.036)*	*(0.036)*	*(0.037)*	***(0.041)***	*(0.041)*	*(0.041)*
infl	0.019	**0.007**	0.013	-0.002	0.021	0.009	**0.006**	0.011	0.010
	(0.037)	***(0.038)***	*(0.037)*	*(0.038)*	*(0.037)*	*(0.038)*	***(0.043)***	*(0.043)*	*(0.043)*
intr	0.064	**0.057**	0.053	0.044	0.061	0.056	**0.012**	0.012	0.010
	(0.043)	***(0.044)***	*(0.043)*	*(0.044)*	*(0.043)*	*(0.044)*	***(0.049)***	*(0.050)*	*(0.050)*
unpl	-0.144***	**-0.100** **	-0.123***	-0.079*	-0.146***	-0.102**	**-0.104** **	-0.086*	-0.107*
	(0.044)	***(0.044)***	*(0.044)*	*(0.045)*	*(0.044)*	*(0.044)*	***(0.049)***	*(0.050)*	*(0.049)*
F(6,513)	48***	**42*** **	50***	43***	48***	41***	**16*** **	16***	16***
adjusted R^2	0.352	**0.317**	0.361	0.326	0.353	0.318	**0.148**	0.144	0.148

Table SI.2: Tests for bi-monthly changes in fine-grained (200 bins based) complexity in form predictable information (E) and predictive complexity (C), measured according to frequency counts ($_{fq}$) and ϵ-machines ($_{eM}$); showing unstandardized beta coefficients, with standard errors in italic parentheses. *** p<0.01, ** p<0.05, * p<0.1 (N = 520).

	(1)	(2)	(3)	(4)	(5)	(6)	(7)	(8)	(9)
Dep. Var.	E_{fq_200}	**E_{eM_200}**	E_{fq_200}	E_{eM_200}	E_{fq_200}	E_{eM_200}	**C_{eM_200}**	C_{eM_200}	C_{eM_200}
constant	0.008<br>*(0.036)*	**0.017**<br>***(0.035)***	0.008<br>*(0.036)*	0.017<br>*(0.035)*	0.008<br>*(0.036)*	0.017<br>*(0.035)*	**0.006**<br>***(0.037)***	0.005<br>*(0.037)*	0.006<br>*(0.037)*
AT_{emp}	0.094**<br>*(0.043)*	**0.077***<br>***(0.042)***					**0.122*****<br>***(0.045)***		
AT_{lin}			0.076*<br>*(0.045)*	0.061<br>*(0.044)*				0.163***<br>*(0.047)*	
AT_{exp}					0.093**<br>*(0.043)*	0.078*<br>*(0.041)*			0.128***<br>*(0.044)*
dep_{t-1}	0.510***<br>*(0.038)*	**0.568*****<br>***(0.036)***	0.516***<br>*(0.038)*	0.572***<br>*(0.036)*	0.510***<br>*(0.038)*	0.568***<br>*(0.036)*	**0.507*****<br>***(0.038)***	0.495***<br>*(0.038)*	0.505***<br>*(0.038)*
GDPr	0.040<br>*(0.037)*	**0.032**<br>***(0.035)***	0.044<br>*(0.037)*	0.035<br>*(0.035)*	0.040<br>*(0.037)*	0.032<br>*(0.035)*	**0.014**<br>***(0.038)***	0.015<br>*(0.037)*	0.013<br>*(0.038)*
infl	0.001<br>*(0.039)*	**-0.015**<br>***(0.037)***	-0.005<br>*(0.038)*	-0.020<br>*(0.037)*	0.000<br>*(0.038)*	-0.016<br>*(0.037)*	**-0.020**<br>***(0.039)***	-0.014<br>*(0.039)*	-0.020<br>*(0.039)*
intr	0.057<br>*(0.045)*	**0.029**<br>***(0.043)***	0.052<br>*(0.045)*	0.025<br>*(0.043)*	0.058<br>*(0.045)*	0.030<br>*(0.043)*	**0.044**<br>***(0.045)***	0.056<br>*(0.045)*	0.048<br>*(0.046)*
unpl	-0.109**<br>*(0.045)*	**-0.073***<br>***(0.043)***	-0.110**<br>*(0.046)*	-0.074*<br>*(0.044)*	-0.108**<br>*(0.045)*	-0.072*<br>*(0.042)*	**-0.016**<br>***(0.045)***	-0.037<br>*(0.046)*	-0.016<br>*(0.045)*
$F(6,513)$	41***	**49*****	40***	48***	41***	49***	**36*****	37***	36***
adjusted R^2	0.314	**0.356**	0.312	0.354	0.314	0.356	**0.287**	0.293	0.289

SI.4. Full Array of Models for H2

Model (2) in Tables SI.3 and SI.4 is the one presented in the main article. As can be seen, it is among those models in which algorithmic trading has the least influence, and are therefore a rather conservative estimate of our broader results.

Table SI.3: Tests for bi-monthly changes in coarse-grained (20 bins based) remaining uncertainty in form entropy rate (h), measured according to frequency counts ($_{fq}$) and ϵ-machines ($_{eM}$); showing unstandardized beta coefficients, with standard errors in italic parentheses. *** p<0.01, ** p<0.05, * p<0.1 (N = 520).

	(1)	(2)	(3)	(4)	(5)	(6)
Dep. Var.	h_{fq_20}	$\mathbf{h_{eM\_20}}$	h_{fq_20}	h_{eM_20}	h_{fq_20}	h_{eM_20}
constant	-0.011<br>*(0.040)*	**-0.014**<br>***(0.041)***	-0.010<br>*(0.041)*	-0.013<br>*(0.041)*	-0.011<br>*(0.040)*	-0.014<br>*(0.041)*
AT_{emp}	-0.262***<br>*(0.048)*	**-0.209*****<br>***(0.049)***				
AT_{lin}			-0.248***<br>*(0.051)*	-0.187***<br>*(0.052)*		
AT_{exp}					-0.262***<br>*(0.048)*	-0.206***<br>*(0.049)*
dep_{t-1}	0.126***<br>*(0.045)*	**0.210*****<br>***(0.044)***	0.134***<br>*(0.045)*	0.219***<br>*(0.044)*	0.127***<br>*(0.045)*	0.212***<br>*(0.044)*
GDPr	-0.051<br>*(0.041)*	**-0.030**<br>***(0.042)***	-0.058<br>*(0.041)*	-0.035<br>*(0.042)*	-0.051<br>*(0.041)*	-0.030<br>*(0.042)*
infl	0.040<br>*(0.043)*	**0.006**<br>***(0.044)***	0.049<br>*(0.043)*	0.016<br>*(0.044)*	0.045<br>*(0.042)*	0.010<br>*(0.043)*
intr	0.040<br>*(0.049)*	**0.001**<br>***(0.050)***	0.042<br>*(0.050)*	0.006<br>*(0.051)*	0.037<br>*(0.049)*	-0.001<br>*(0.050)*
unpl	-0.098**<br>*(0.049)*	**-0.066**<br>***(0.050)***	-0.081<br>*(0.050)*	-0.055<br>*(0.051)*	-0.102**<br>*(0.049)*	-0.070<br>*(0.050)*
F(6,513)	17***	**13*****	16***	12***	17***	13***
adjusted R^2	0.157	**0.119**	0.148	0.111	0.158	0.119

Table SI.4: Tests for bi-monthly changes in fine-grained (200 bins based) remaining uncertainty in form entropy rate (h), measured according to frequency counts (fq) and ϵ-machines (eM); showing unstandardized beta coefficients, with standard errors in italic parentheses. *** p<0.01, ** p<0.05, * p<0.1 (N = 520).

	(1)	(2)	(3)	(4)	(5)	(6)
Dep. Var.	h_{fq_200}	$\mathbf{h_{eM\_200}}$	h_{fq_200}	h_{eM_200}	h_{fq_200}	h_{eM_200}
constant	0.002	**0.001**	0.000	-0.001	0.002	0.001
	(0.036)	***(0.036)***	*(0.036)*	*(0.036)*	*(0.036)*	*(0.036)*
AT_{emp}	0.088**	**0.076***				
	(0.043)	***(0.043)***				
AT_{lin}			0.152***	0.119***		
			(0.045)	*(0.045)*		
AT_{exp}					0.096**	0.082*
					(0.042)	*(0.042)*
dep_{t-1}	0.566***	**0.570*****	0.547***	0.559***	0.563***	0.568***
	(0.037)	***(0.036)***	*(0.037)*	*(0.037)*	*(0.037)*	*(0.036)*
GDPr	0.010	**0.005**	0.010	0.005	0.010	0.005
	(0.036)	***(0.036)***	*(0.036)*	*(0.036)*	*(0.036)*	*(0.036)*
infl	0.008	**0.007**	0.020	0.015	0.009	0.007
	(0.038)	***(0.038)***	*(0.038)*	*(0.038)*	*(0.038)*	*(0.038)*
intr	0.017	**0.045**	0.034	0.057	0.020	0.047
	(0.044)	***(0.044)***	*(0.044)*	*(0.044)*	*(0.044)*	*(0.044)*
unpl	0.016	**0.024**	-0.007	0.007	0.016	0.024
	(0.043)	***(0.043)***	*(0.044)*	*(0.044)*	*(0.043)*	*(0.043)*
$F(6,513)$	46***	**45*****	47***	46***	46***	45***
adjusted R^2	0.340	**0.337**	0.349	0.342	0.341	0.338

SI.5. Histograms of Bid-Ask Spreads

Figure SI.3 shows the raw (non-standardized) bid-ask spreads for our eight currency pairs. The mean and standard deviation naturally varies, depending on the relative value of the currency.

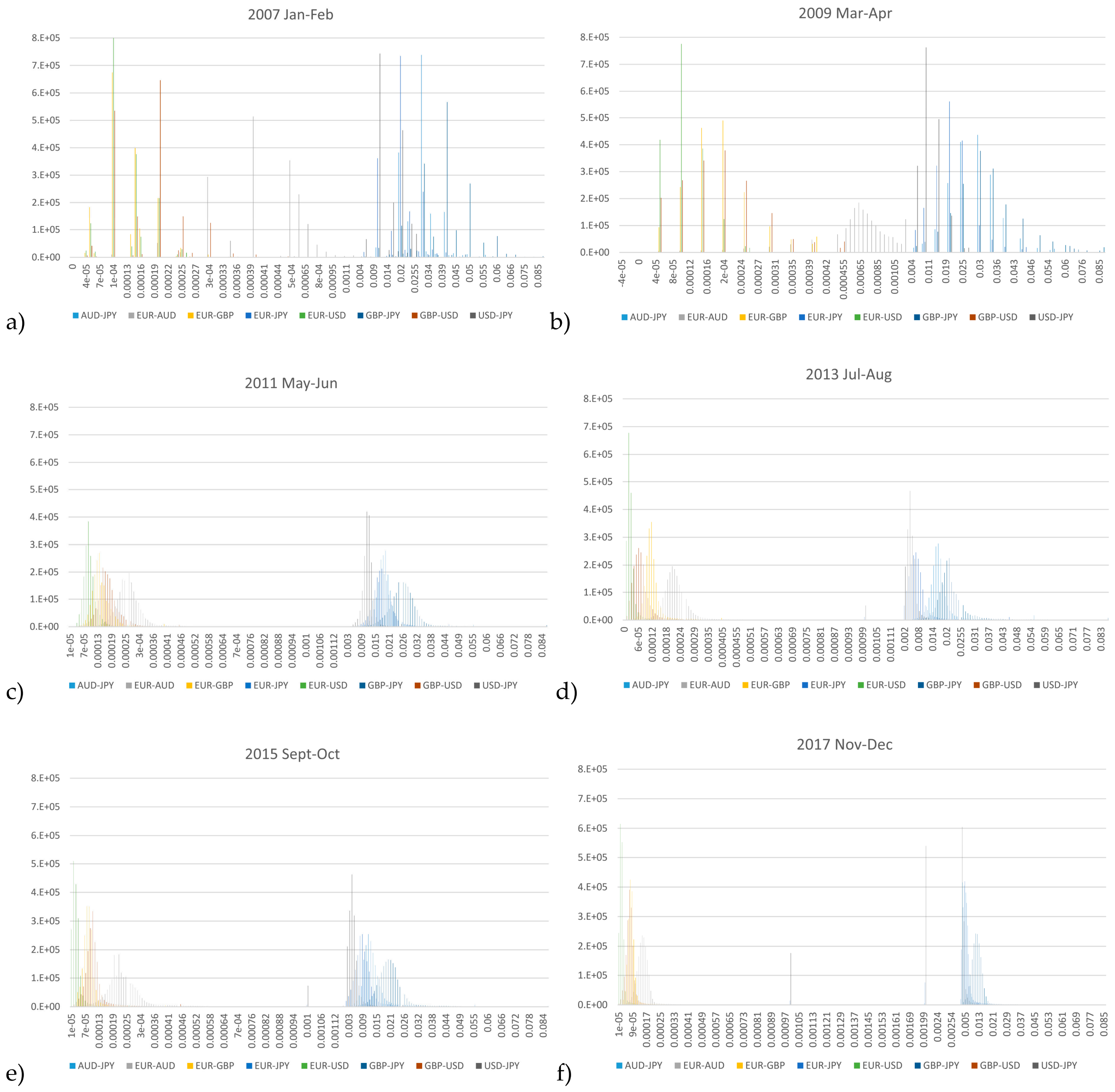

Figure SI.3 Histograms of unbinned bid-ask spreads for (a) Jan-Feb 2007; (b) Mar-Apr 2009; (c) May-Jun 2011; (d) Jul-Aug 2013; (e) Sep-Oct 2015; (f) Nov-Dec 2017.

Figure SI.4 shows the standardized bid-ask spreads of all eight currency pairs together, standardized by subtracting the respective mean and dividing it by its standard deviation. It is noticeable that the unbinned distribution became more uniform (less extreme events), which is then expressed in increased entropy (uncertainty) in our analysis.

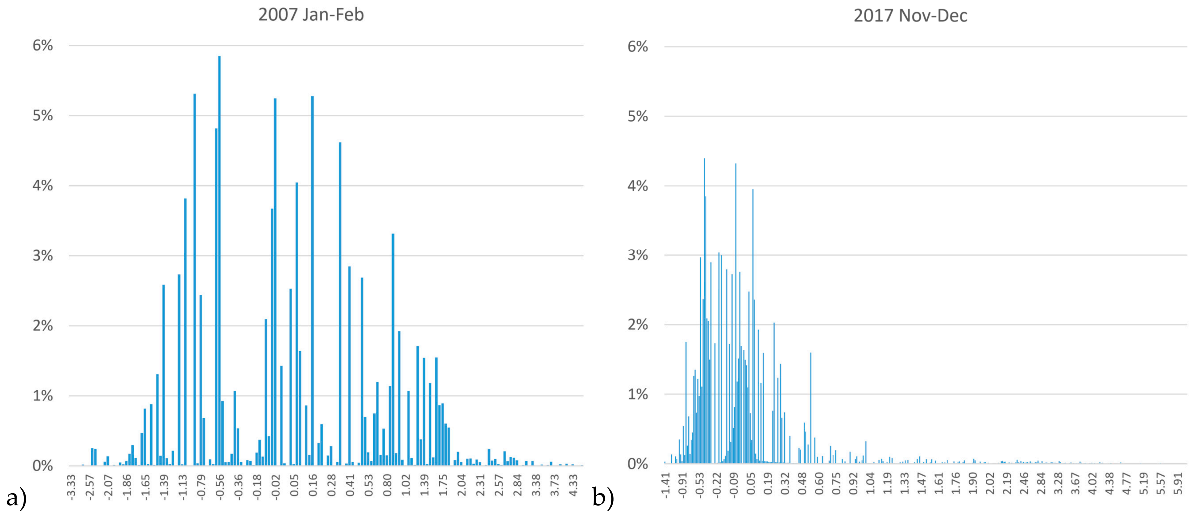

Figure SI.4 Histograms of standardized bid-ask spreads for (a) Jan-Feb 2007; (b) Nov-Dec 2017.

SI.6. Conditional Entropy Plots

In line with the chain rule of entropy, Figure SI.5 shows the diverging uncertainty between the more coarse-grained and the more fine-grained perspective. In 2007, both were still similar. The area between both levels of uncertainty is the conditional uncertainty (conditioned on the more coarse-grained resolution level).

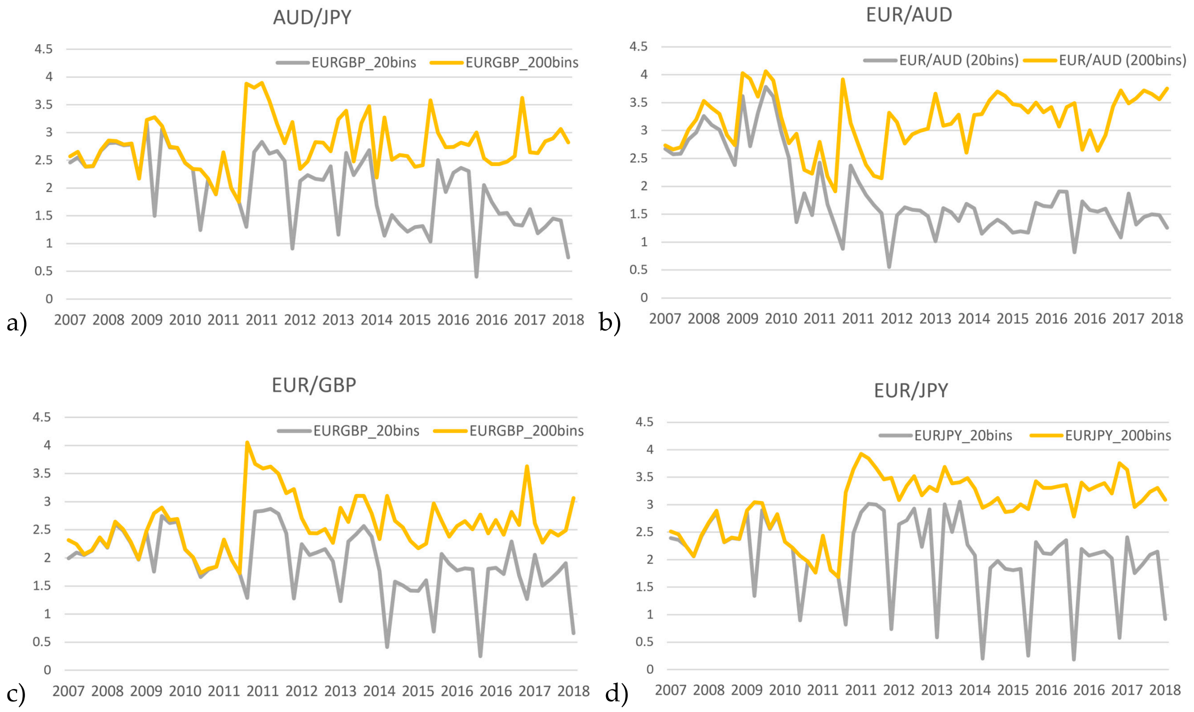

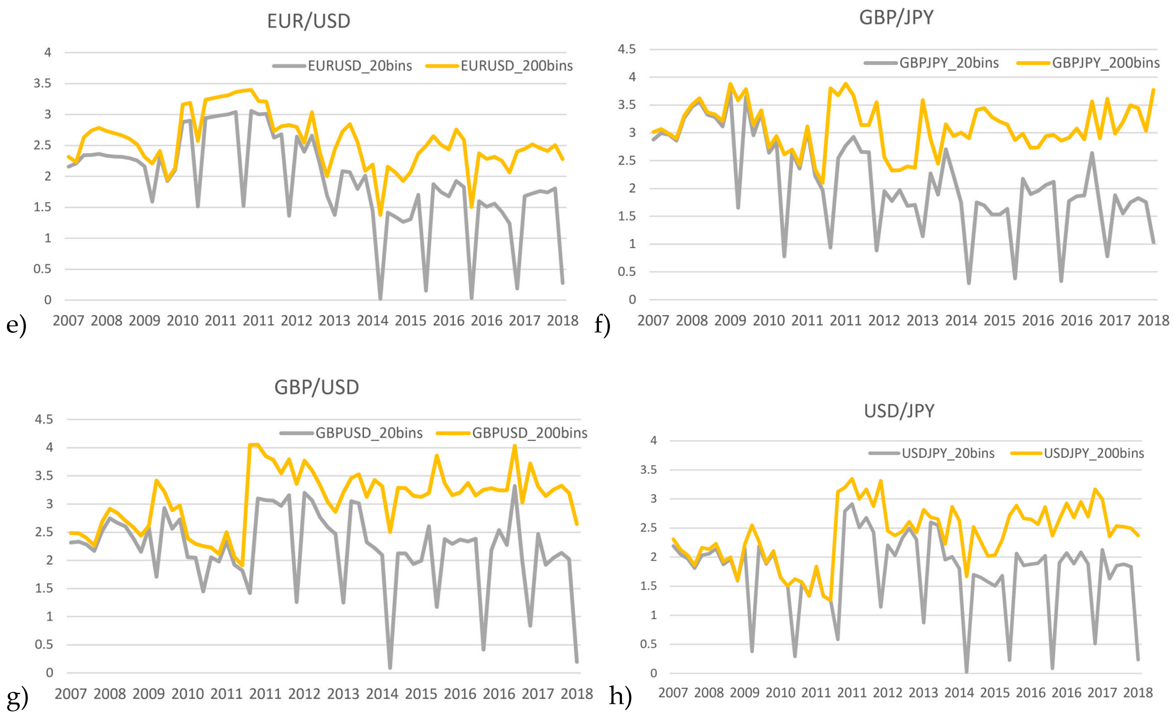

Figure SI.5 Conditional entropy plots for all currencies.

SI.7. Decreasing Bid-Ask Spreads

Gaining some intuition about the evolution of bid-ask spreads, Figure SI.6 shows visual evidence for a changing bid-ask spreads over the decade. In agreement with Hendershott and Moulto [6] the descriptive data shows an increase of the spread during the 2008 financial crisis (Lehman Brothers filed bankruptcy in September 2008) (in 2011, Hendershott et al. [7] speculated that there could be increases of temporary nature regarding realized spreads, related to asymmetries exploited by liquidity suppliers during early phases of algorithmic trading. From a decade long perspective, this might be the case, but it also seems to be the case that the increase in spread rather is linked to the financial crisis and its accompanying turmoil per se). Over the decade, however, bi-monthly bid-ask spreads decreased by half, sometimes more. In Jan-Feb 2007, the average bid-ask spread of EUR/USD was precisely one-hundredth of a cent higher than in Nov-Dec 2017 (0.00013 vs. 0.00003). Figure SI.6b shows that a similar decreasing tendency also applies to the bi-monthly standard deviation of the bid-ask spread over the same period.

It is important to point out that our relatively short time window cannot eliminate the possibility that the rather large and volatile bid-ask spread around 2008-2009 is rather the result of the global financial crisis.

Our analysis of predicting the bi-monthly means and standard deviations with our six IVs (Table SI.5), confirms that both decreases are linked to a strongly and monotonically increasing tendency that is in line with the rise of algorithmic trading (our independent variable AT, in its three different versions, namely empirical (AT_{emp}), linear (AT_{lin}) and exponential (AT_{exp})). The strongest predictor is the lagged path dependency term, closely followed by our AT variable. Interest rate and unemployment rate are also significant predictors, but less important in terms of effect size. GDP growth rate and inflation, which have seen important variances over the decade, do not play a significant role in predicting changing bid-ask tendencies. Additionally, to the negative association between algorithmic trading and bid-ask spread, we

can also add that the standard deviation decreased in association with our increased algorithmic trading tendency, which gives us first indications in terms of temporal predictability.

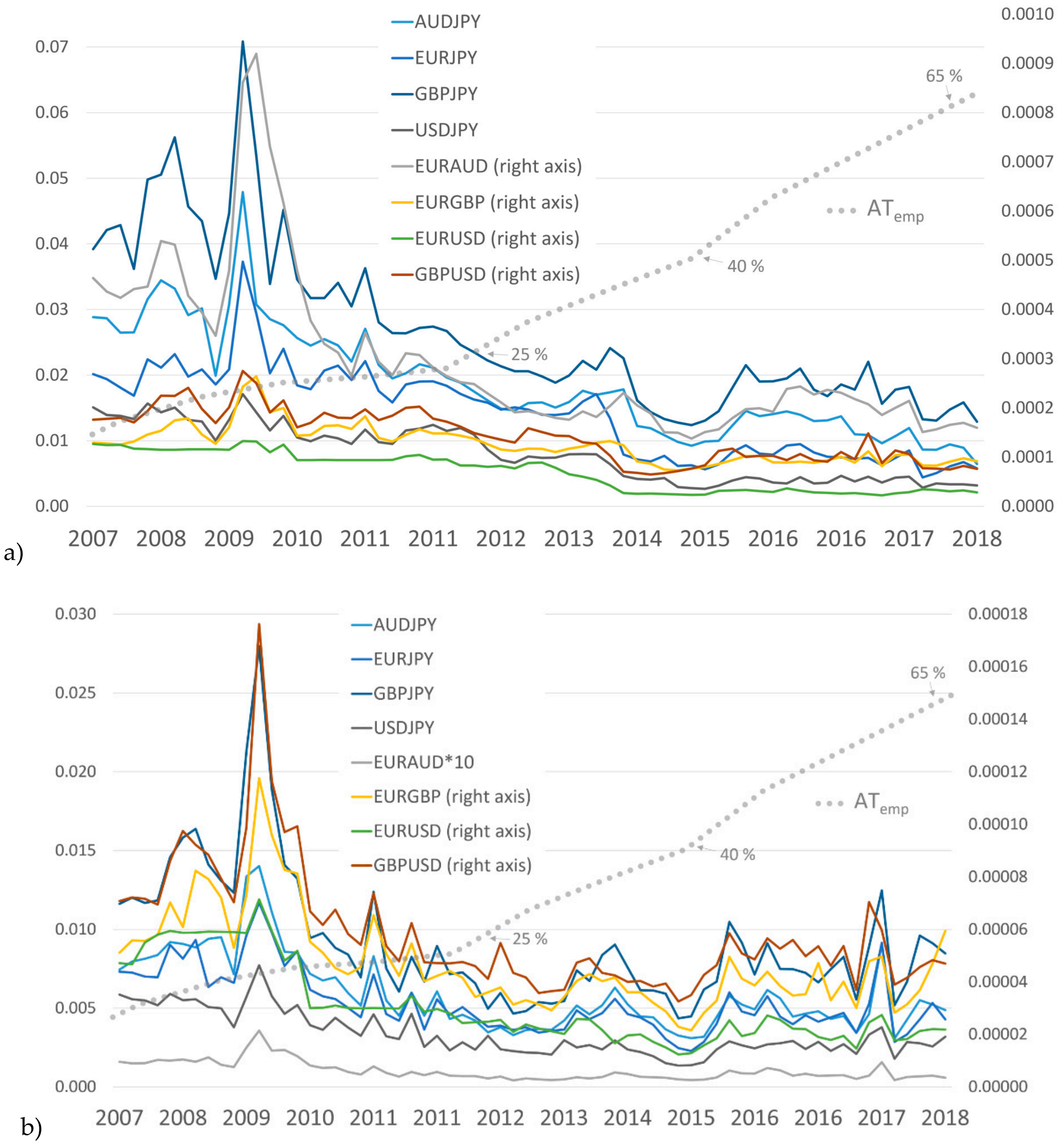

Figure SI.6 Bi-monthly bid-ask spreads Jan 2007 - Dec 2017: (a) mean; (b) standard deviation. Dotted line shows algorithmic trading empirical average with linear extrapolation from Figure 3 (scale indicated by inserted % labels).

Table SI.5: Tests for bi-monthly changes in mean (M) and standard deviation (St) of bid-ask spread; showing unstandardized beta coefficients, with standard errors in italic parentheses. *** p<0.01, ** p<0.05, * p<0.1 (N = 520). .

	**(1)**	**(2)**	**(3)**	**(4)**	**(5)**	**(6)**
Dep. Var.	M	St	M	St	M	St
constant	-0.022	-0.013	-0.018	-0.010	-0.022	-0.013
	(0.018)	*(0.026)*	*(0.017)*	*(0.025)*	*(0.028)*	*(0.026)*
AT_{emp}	-0.194***	-0.099***				
	(0.029)	*(0.033)*				
AT_{lin}			-0.276***	-0.189***		
			(0.032)	*(0.036)*		
AT_{exp}					-0.197***	-0.108***
					(0.029)	*(0.033)*
dep_{t-1}	0.690***	0.686***	0.622***	0.632***	0.684***	0.680***
	(0.032)	*(0.032)*	*(0.034)*	*(0.034)*	*(0.032)*	*(0.033)*
GDPr	0.001	0.035	-0.004	0.039	0.001	0.036
	(0.018)	*(0.026)*	*(0.017)*	*(0.026)*	*(0.018)*	*(0.026)*
infl	-0.003	-0.023	-0.003	-0.036	0.001	-0.023
	(0.019)	*(0.027)*	*(0.018)*	*(0.027)*	*(0.019)*	*(0.027)*
intr	0.061***	0.070**	0.058***	0.056*	0.058***	0.067**
	(0.022)	*(0.031)*	*(0.021)*	*(0.031)*	*(0.022)*	*(0.031)*
unpl	-0.070***	-0.068**	-0.056***	-0.055*	-0.074***	-0.070**
	(0.022)	*(0.032)*	*(0.022)*	*(0.032)*	*(0.022)*	*(0.032)*
$F(6,513)$	453***	167***	481***	176***	455***	168***
adjusted R^2	0.839	0.658	0.847	0.669	0.84	0.659

References

1. Allison, P. *Statistical Horizons*. June 2, 2015,.
2. Darmon, D. Statistical Methods for Analyzing Time Series Data Drawn from Complex Social Systems. *PhD Thesis, University of Maryland* **2015**, *Supervised by Michelle Girvan and William Rand*, doi:10.13016/M2V93N.
3. James, R.G.; Ellison, C.J.; Crutchfield, J.P. dit: a Python package for discrete information theory. *Journal of Open Source Software* **2018**, *3*, 738.
4. Crutchfield, J.P. Between order and chaos. *Nat Phys* **2012**, *8*, 17–24, doi:10.1038/nphys2190.
5. Shalizi, C.R.; Crutchfield, J.P. Computational Mechanics: Pattern and Prediction, Structure and Simplicity. *Journal of Statistical Physics* **2001**, *104*, 817–879, doi:10.1023/A:1010388907793.
6. Hendershott, T.; Moulton, P.C. Automation, speed, and stock market quality: The NYSE's Hybrid. *Journal of Financial Markets* **2011**, *14*, 568–604, doi:10.1016/j.finmar.2011.02.003.
7. Hendershott, T.; Jones, C.M.; Menkveld, A.J. Does Algorithmic Trading Improve Liquidity? *The Journal of Finance* **2011**, *66*, 1–33, doi:10.1111/j.1540-6261.2010.01624.x.
